# Identification of lactylation-related biomarkers in osteoporosis from transcriptome and single-cell data

**DOI:** 10.3389/fendo.2025.1621878

**Published:** 2025-08-25

**Authors:** Jiafeng Peng, Hongxing Zhang, Huaize Wang, Ting Jiang, Minglei Gao, Xingfu Ma, Yingzong Xiong, Yingchun Li, Ran Xu, Junchen Zhu

**Affiliations:** ^1^ Department of Orthopedics I, Second Affiliated Hospital, Anhui University of Traditional Chinese Medicine, Hefei, Anhui, China; ^2^ Department of Spinal Surgery, First Affiliated Hospital, Anhui University of Traditional Chinese Medicine, Hefei, China; ^3^ Graduate School, Anhui University of Traditional Chinese Medicine, Hefei, China

**Keywords:** osteoporosis, lactylation, biomarkers, nomogram, single-cell RNA sequencing

## Abstract

**Background:**

Emerging evidence indicates that lactase-mediated histone lactylation can activate osteogenic gene expression and promote bone formation. However, the role of lactylation-related genes (LRGs) in osteoporosis (OP) remains unclear. This study aims to clarify the key roles of LRGs and the molecular mechanisms of related biomarkers in OP.

**Methods:**

Three datasets (GSE7158, GSE56815, and GSE147287) and 327 LRGs were analyzed in this study. First, the biomarkers associated with OP were identified through differential gene expression analysis, machine learning algorithms, expression validation, and receiver operating characteristic (ROC) curve analysis. Subsequently, nomograms, functional enrichment analyses, immune infiltration analyses, regulatory network construction, drug prediction, and molecular docking were performed to characterize the functional and clinical significance of the biomarkers. Single-cell analysis was used to screen key cell types. Finally, reverse transcription quantitative polymerase chain reaction (RT-qPCR) was conducted to validate biomarker expression.

**Results:**

CSRP2 and FUBP1 can serve as biomarkers for the early prediction of osteoporosis risk in individuals with low peak bone mass or bone mineral density. The nomogram showed that these two biomarkers could accurately predict OP risk. Functional analysis revealed that *CSRP2* and *FUBP1* were closely associated with inflammation regulation. FUBP1 was strongly positively correlated with mesenchymal stem cells (MSCs). Both CSRP2 and FUBP1 exhibited strong binding to bisphenol A and tetrachlorodibenzodioxin, with binding energies < −5 kcal/mol. The key cell types associated with OP were identified as bone marrow MSCs, T cells, natural killer cells, and hematopoietic stem cells. *CSRP2* expression was significantly associated with natural killer cell differentiation. RT-qPCR confirmed that *CSRP2* was downregulated and *FUBP1* was upregulated in OP samples, consistent with the findings in the GSE7158 and GSE56815 datasets.

**Conclusions:**

CSRP2 and FUBP1 can serve as biomarkers for the early prediction of osteoporosis risk in individuals with low BMD/PBM. The findings of this study offer critical clinical guidance for OP prevention and treatment.

## 1 Introduction

Osteoporosis (OP) is a systemic skeletal disorder characterized by reduced bone mass and microarchitectural deterioration of bone tissue, leading to compromised bone strength and a marked increase in fracture susceptibility (1, 2). OP pathogenesis primarily involves an imbalance between osteoclast-mediated bone resorption and osteoblast-driven bone formation (3), with various contributing factors, including immune dysregulation, genetic predisposition, aging, and smoking, which can synergistically accelerate bone deterioration (2). Current clinical management of OP primarily involves pharmacological interventions, especially anti-resorptive agents and anabolic therapies, which target bone remodeling regulation (4). However, anti-resorptive agents such as bisphosphonates demonstrate limited efficacy in preventing non-vertebral fractures (5), while anabolic agents are subject to restricted treatment durations and require sequential therapeutic regimens to sustain clinical benefits (4). Emerging mesenchymal stem cell (MSC) therapies inhibit OP through paracrine growth factor secretion and bone remodeling regulation (6); however, the current MSC strategies do not address core metabolic dysfunctions and can lead to adverse effects (7). Current biomarkers for OP include established bone turnover markers, comprising bone formation and bone resorption markers (8), alongside novel emerging candidates. Procollagen type I N-terminal propeptide (PINP) and bone-specific alkaline phosphatase (BALP) (9) are key bone formation markers that offer distinct clinical profiles. PINP is an internationally recognized cornerstone indicator for predicting fracture risk and monitoring anabolic therapies, like teriparatide, demonstrating rapid responsiveness within weeks to months; however, its accuracy is compromised by renal impairment and is prone to non-specific elevation during hepatic dysfunction or inflammatory states (10). Conversely, BALP exhibits higher bone tissue specificity with minimal interference from hepatic dysfunction, providing a more reliable assessment in chronic kidney disease (CKD) patients; however, its clinical application is hindered by limited assay standardization, which results in significant inter-laboratory variability (11). β-Isomerized C-terminal telopeptide of type I collagen (β-CTX) is a key bone resorption marker that serves as an internationally recommended metric. It demonstrates high sensitivity for monitoring antiresorptive therapy efficacy (e.g., bisphosphonates), typically exhibiting significant decreases in serum β-CTX concentrations within 3–6 months, making it valuable for assessing treatment adherence. However, its measurement requires a strict fasting regimen due to susceptibility to dietary interference. β-CTX levels are also significantly impacted by renal impairment, with increased false-positive risk in CKD populations (12).

Novel biomarker development focuses on early or more precise risk stratification. However, studies on OP-related post-translational biomarkers and their pathophysiological regulatory networks are limited. Therefore, there is an urgent need to identify novel biomarkers that can serve as potential therapeutic targets for treating OP. Elucidating the specific regulatory mechanisms of these biomarkers within OP pathogenesis is fundamental for advancing therapeutic strategies through precision medicine targeting. Circulating osteoclast precursor cells (cOCPs) can be used to directly assess osteoclastogenic potential. Compared to dual-energy X-ray absorptiometry, cOCP analysis can provide more economical screening and facilitate early high-risk individual identification. However, due to predominantly small-sample studies, cOCP analysis requires broader validation and assay standardization before clinical implementation (13).

Lactate is a terminal metabolite of glycolysis and functions as a pivotal bioenergetic intermediary in cellular metabolism (14). In 2019, Zhang et al. (15) identified histone lactylation as a novel post-translational modification. Lactate molecules covalently conjugate to lysine residues within both histone and non-histone proteins, regulating chromatin structural remodeling and transcriptional modulation to mediate immunological regulation, metabolic-epigenetic coupling, and pathological microenvironment reprogramming (14). Wu et al. (16) demonstrated that endothelial cell-derived lactate, generated through glycolysis, induces histone H3K18 lactylation in bone marrow MSCs (BM-MSCs), thereby activating osteogenic gene expression programs to potentiate bone formation. Despite these advances, the regulatory mechanisms underlying lactylation modifications in OP remain to be fully elucidated.

**Figure 1:**
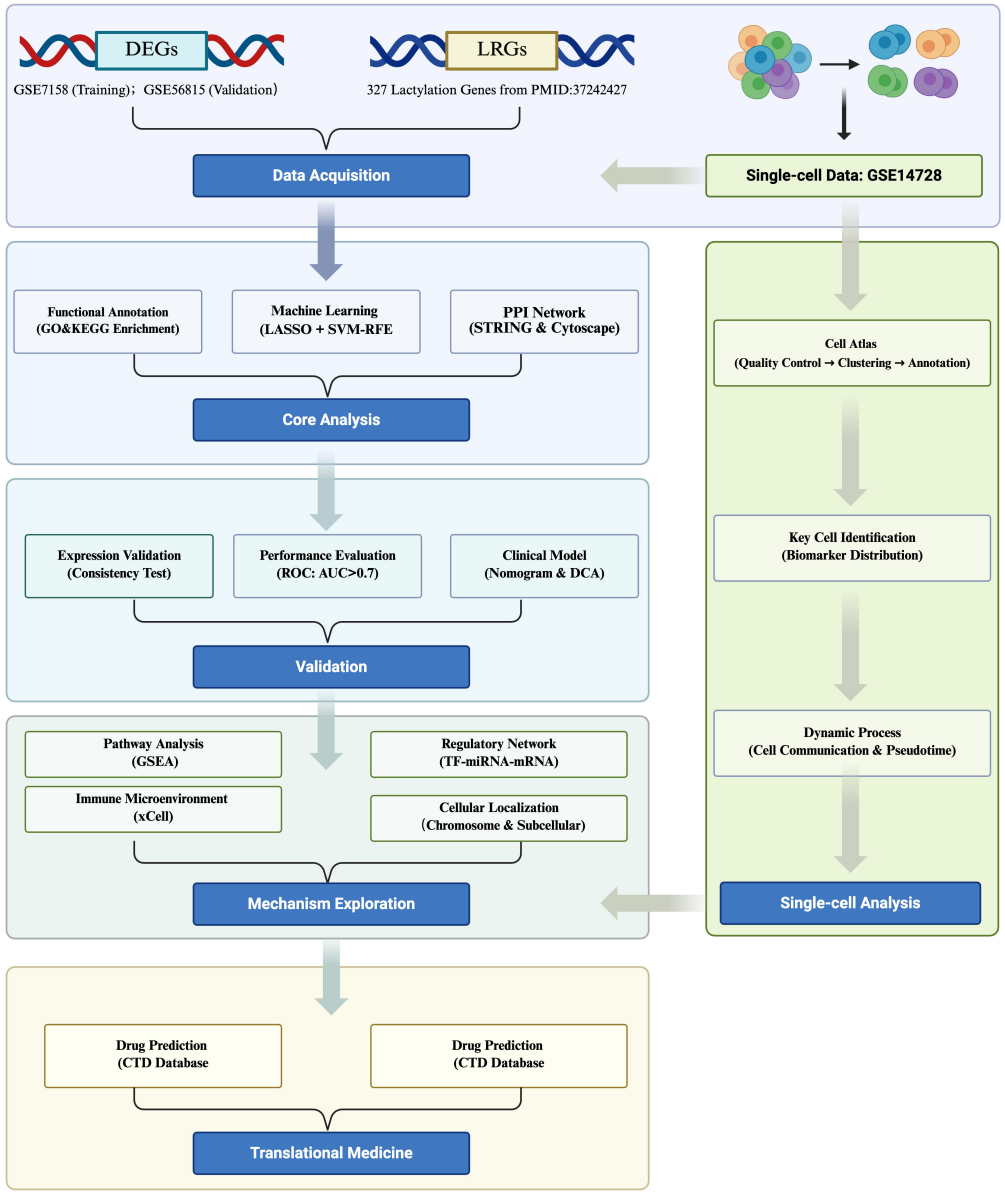
Comprehensive workflow of lactylation biomarker discovery in osteoporosis.

In this study, publicly available transcriptomic and single-cell datasets of OP were used to identify lactylation-modified biomarkers through an integrated analytical pipeline comprising differential expression analysis, machine learning algorithms, and multi-cohort expression validation. Subsequent multidimensional analyses, encompassing nomogram construction, functional enrichment profiling, immune infiltration assessment, molecular network reconstruction, computational drug prediction, and molecular docking simulations, were systematically conducted to decipher biomarker-mediated OP pathogenesis. Single-cell transcriptomic analysis identified critical cell populations and delineated biomarker expression dynamics at cellular resolution, which were cross-validated through clinical specimens to confirm pathological relevance. This multilayered approach elucidates the molecular pathophysiology of OP biomarkers while establishing novel therapeutic target discovery pipelines, thereby advancing mechanistic insights into OP pathogenesis. A schematic overview of the integrated analytical pipeline is provided in **Figure 1**.

## 2 Materials and methods

### 2.1 Data source

Three datasets related to OP (GSE7158, GSE56815, and GSE147287) were obtained from the Gene Expression Omnibus (GEO) database (http://www.ncbi.nlm.nih.gov/geo/; accessed on November 27, 2024) (17). The GSE7158 (GPL570) dataset, utilized as the training set, included monocyte samples isolated from the peripheral blood of 12 subjects with low peak bone mass (PBM; high OP risk) and 14 subjects with high PBM (control) (18). The GSE56815 (GPL96) dataset, utilized as the validation set, included monocyte samples isolated from the whole blood of 40 subjects with low bone mineral density (BMD; high OP risk) and 40 subjects with high BMD (control) (19). Although low PBM and BMD are established risk factors for OP, they do not alone constitute a formal clinical diagnosis of OP according to the current guidelines (20). These cohorts represent individuals with significantly impaired bone health, characteristic of OP susceptibility. Lastly, the GSE147287 (GPL24676) dataset included the single-cell RNA sequencing (scRNA-seq) data of CD271+ sorted mesenchymal stem cells and their progeny​ from OP patient bone marrow (21, 22). In addition, 327 lactylation-related genes (LRGs) were retrieved from previous literature (**Supplementary Table S1**) (23).

### 2.2 Recognition of candidate genes

The limma (v3.54.0) package was utilized to identify differentially expressed genes (DEGs) between the OP and control groups within the GSE7158 dataset (|log2fold-change (FC)| > 0.5 and *P* < 0.05). Additionally, the ggplot2 (v 3.4.1) package (24) and ComplexHeatmap (v2.14.0) (25) package were utilized to generate the volcano plot and heatmap of the DEGs, respectively. The VennDiagram (v1.7.1) package (26) was used to identify the overlapping LRGs and DEGs (candidate genes).

### 2.3 Functional analysis of candidate genes

The candidate genes were subjected to Gene Ontology (GO) and Kyoto Encyclopedia of Genes and Genomes (KEGG) enrichment analyses via the clusterProfiler (v4.2.2) package (27) (*P* < 0.05). The protein–protein interaction (PPI) network was assembled using the STRING database (https://www.string-db.org) at a default confidence threshold of > 0.4 (28), and this threshold could balance the network coverage and reliability and illustrated using Cytoscape (v3.8.2) (29).

### 2.4 Identification of biomarkers

The least absolute shrinkage and selection operator (LASSO) analysis of the candidate genes was performed using the glmnet (v4.1.4) package (30) under the following parameters: standardize = True, alpha = 1, family = binomial, and nfolds = 5. Genes with scores > 0 were regarded as the LASSO genes. Additionally, the transcriptomic data were subjected to LASSO analysis to eliminate redundant features and obtain key genes associated with OP and lactylation modification, thereby avoiding overfitting. The support vector machine-recursive feature elimination (SVM-RFE) analysis was conducted using the caret (v6.0.93) package (31) to determine the accuracy and error rate of each feature subset; the subset with the highest accuracy rate was selected to identify the SVM-RFE genes. The SVM-RFE analysis recursively eliminated features and retained the subset of features that contributed significantly to the classification of the OP and the control group. This method is suitable for high-dimensional, small-sample data and can capture nonlinear relationships. The feature genes were obtained by intersecting the selected LASSO and SVM-RFE genes using the VennDiagram (v1.7.1) package. Subsequently, the expression of these feature genes in the GSE7158 and GSE56815 datasets was analyzed utilizing the Wilcoxon test and visualized using the ggplot2 (v3.4.1) package to identify candidate biomarkers (*P* < 0.05). Finally, the receiver operating characteristic (ROC) curves of the candidate biomarkers in the GSE7158 dataset were generated utilizing the pROC (v 1.18.0) package (32) to identify the key biomarkers (area under the curve, AUC > 0.7). Subsequently, a correlation analysis was conducted between the expression of these biomarkers and PBM/BMD. In addition, chromosomal and subcellular localization analyses were conducted using the RCircos (v1.2.2) package (33) and GeneCard database (https://www.genecards.org/) to determine the chromosomal and subcellular locations of the biomarkers, respectively.

### 2.5 Nomogram construction and evaluation

A nomogram was constructed based on the biomarkers in the GSE7158 dataset using the rms (v6.5.0) package (34) to examine their reliability in predicting OP onset. Thereafter, a calibration curve was plotted using the rms (v 6.5.0) package, and the Hosmer–Lemeshow (HL) test was conducted (*P* > 0.05). Decision curve analysis (DCA) was performed using the rmda (v 1.6) package (35) to evaluate the effectiveness of the nomogram.

### 2.6 Gene set enrichment analysis

The biomarkers in the GSE7158 dataset were subjected to GSEA, and the c2.cp.kegg.v7.4.symbols.gmt data of the GSE7158 dataset was derived from the Molecular Signatures Database (MSigDB) (https://www.gsea-msigdb.org/gsea/msigdb/). The Spearman correlation between the biomarkers and other genes was determined using the psych (v2.1.6) package (36). Subsequently, GSEA was conducted for each key gene using the clusterProfiler (v 4.2.2) package (|normalized enrichment score| > 1, Padj < 0.05), and the top 10 pathways were illustrated utilizing the enrichplot (v1.18.3) package (37). Lastly, the GeneMANIA database (http://genemania.org) was utilized to anticipate the genes associated with the biomarkers and their associated functions, and a gene–gene interaction (GGI) network was developed.

### 2.7 Immune infiltration analysis

The relative abundance of 64 types of immune cells in the GSE7158 dataset was calculated using the xCell (v 1.1.0) package (38). The Wilcoxon test was employed to assess the differences in the infiltration of these immune cell types between the OP and control groups (*P* < 0.05) in the GSE7158 dataset, and the results were illustrated using the ggplot2 (v3.4.1) package to screen for differential immune cells. Subsequently, Spearman correlation analysis was performed utilizing the psych (v2.1.6) software package to explore the relationships among the differential immune cells, as well as the relationships between the differential immune cells, biomarkers, and MSCs (|correlation coefficients| > 0.30, *P* < 0.05).

### 2.8 Construction of a regulatory network

To probe the molecular regulatory mechanisms of the biomarkers, microRNAs (miRNAs) acting on the biomarkers were predicted in the miRDB database (https://mirdb.org/) and the miRTarBase database (https://mirtarbase.cuhk.edu.cn/~miRTarBase/miRTarBase_2025/php/index.php). The VennDiagram (v1.7.1) package was used to identify the overlapping miRNAs from the two databases. Similarly, the transcription factors (TFs) regulating the biomarkers were predicted in the KnockTF database (http://www.licpathway.net/KnockTF/index.php). The miRNA–mRNA and TF–mRNA–miRNA networks were constructed and illustrated using the Cytoscape (v3.8.2) software.

### 2.9 Drug prediction and molecular docking

The biomarker-targeting drugs were identified using the Comparative Toxicogenomics Database (CTD) (https://ctdbase.org/), and the drug–biomarker network was illustrated using the Cytoscape (v3.8.2) software. The three-dimensional (3D) structures of the drugs were derived from the AlphaFold database (https://alphafold.ebi.ac.uk/). The biomarkers were uploaded to the Cavity-detection guided Blind Docking (CB-Dock2) database (https://www.rcsb.org/) to obtain the *.pdbqt format files of their protein 3D structures. Binding energy was obtained via molecular docking. Drug–target combinations with binding energies below −5 kcal/mol were considered to have good binding ability (39). The molecular docking results were illustrated using the Pymol (v2.2.0) software (40).

### 2.10 scRNA-seq analysis

The GSE147287 dataset was analyzed using the Seurat (v4.3.0) package (41) (min.features = 200 and min.cells = 3), and the high-quality cells and genes were selected (200 < nFeature_RNA < 4,000, nCount_RNA < 60,000, and percent.mt < 25%). The filtered single-cell data was standardized using the NormalizeData function, and the top 2,000 highly variable genes (HVGs) were selected using the FindVariableFeatures function in Seurat (v4.3.0). Thereafter, the ScaleData function was used to scale all the genes, and the runPCA function was used to perform principal component (PC) analysis (PCA) on the identified HVGs (*P* < 0.05). The JackStraw function was utilized to assess the contribution of the PCs through significance testing, and the JackStrawPlot function was used to visualize the results. The Elbowplot function was used to appraise the cumulative contribution of the PCs to the overall data variation to evaluate the appropriate number of PCs for downstream analysis, and the results were visualized using a screen plot. The FindNeighbors function was used to evaluate the similarity between cells, and the FindClusters function was used to classify the cells into different cell clusters (resolution = 0.5) based on the t-SNE clustering method. The FindAllMarkers function was used to identify the marker genes of different cell populations, and the classic marker genes in the CellMarker database (http://xteam.xbio.top/CellMarker/) were utilized to annotate each cell population (**Supplementary Table S2**).

### 2.11 Identification of key cells and cell communication analysis

The FeaturePlot function in the Seurat (v4.3.0) package was utilized to analyze the expression distribution of biomarkers across all cell types in the GSE147287 dataset. The DotPlot was utilized to draw bubble plots of biomarker expression in each cell type to identify key cells. Subsequently, the distribution of the key cells was visualized using a t-SNE plot. Thereafter, the DotPlot function was utilized to draw bubble plots of biomarker expression in the key cells, and the FeaturePlot function was utilized to examine the expression distribution of the biomarkers in key cells. Additionally, the cellular communication network among all the annotated cell types was analyzed using the CellChat (v1.6.1) package (42), and the netVisual_bubble function was utilized to determine the interactions between receptors and ligands of all the annotated cell types (*P* < 0.05, log2 mean (Molecule 1 and 2) ≥ 0.1).

### 2.12 Pseudotime analysis

The key cells were extracted from the GSE147287 dataset and subjected to secondary dimensionality reduction and clustering into different subtypes (resolution = 0.5) to explore their state transition process and predict their direction of differentiation. Subsequently, the relationship between the expression changes of biomarkers and the differentiation of key cells was assessed via cell pseudotime trajectory analysis performed using the Monocle (v2.26.0) package (43), and the results were visualized using the plot_cell_trajectory function.

### 2.13 Reverse transcription-quantitative polymerase chain reaction

A total of 10 peripheral blood mononuclear cell (PBMC) samples (from 5 OP patients and 5 control subjects) were collected from the First Affiliated Hospital of Anhui University of Chinese Medicine (Hefei, China). All the participants gave written informed consent, and the study was approved by the Institutional Ethics Committee (approval number: 2024AH-143). Total RNA from the 10 samples was isolated using TRIzol reagent (Ambion, USA) according to the manufacturer’s protocol, and the RNA concentration was quantified using the NanoPhotometer N50. The cDNA synthesis was performed using the SureScript-First-strand-cDNA-synthesis-kit on the S1000TM Thermal Cycler (Bio-Rad, USA). The primer sequences for the RT-qPCR assay are provided in **Supplementary Table S3**. The RT-qPCR assay was performed on the CFX Connect Real-time Quantitative Fluorescence PCR Instrument (Bio-Rad, USA) under the following conditions: pre-denaturation at 95°C for 1 min, followed by 40 cycles of denaturation at 95°C for 20 s, annealing at 55°C for 20 s, and extension at 72°C for 30 s). The relative mRNA quantification was performed using the 2*^−ΔΔCT^* method with *gapdh* as the internal reference. The results from the RT-qPCR assay were exported to Excel and imported into GraphPad Prism 5 for statistical analysis and visualization (*P* < 0.05).

### 2.14 Statistical analysis

The R (v4.2.2) software was utilized to conduct statistical analysis. Differences between two groups were examined via the Wilcoxon test (*P* < 0.05). For the RT-qPCR analysis, the *t* test was employed for statistical comparisons. Notably, **** represented *P* < 0.0001, *** represented *P* < 0.001, ** represented *P* < 0.01, * represented *P* < 0.05, and ns represented *P* > 0.05 (indicating no significant difference).

## 3 Results

### 3.1 Candidate genes and their associated functions

**Figure 2:**
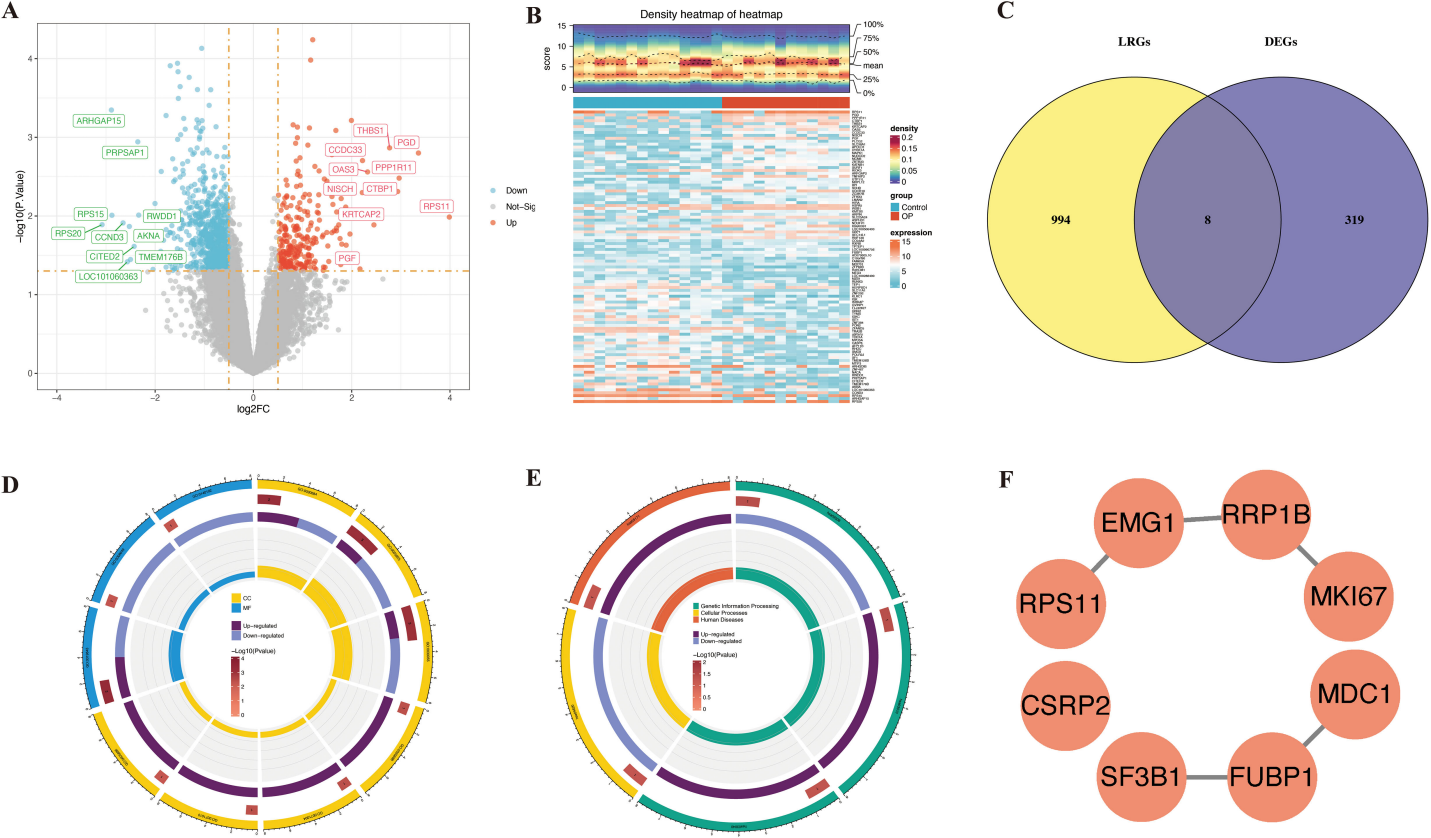
Discovery of lactylation-associated hub genes in osteoporosis and functional insights. ​**(A, B)** Differential expression analysis in GSE7158 dataset visualized by volcano plot **(A)** and heatmap **(B)**. **(C)** Overlap analysis between 1,002 DEGs and 327 lactylation-related genes from public databases. **(D, E)** Functional enrichment of 8 overlapping genes: top GO term **(D)** and KEGG pathway **(E)** shown. **(F)** PPI network construction and hub gene identification.

A total of 1,002 DEGs were identified in the GSE7158 dataset, of which 257 were upregulated and 745 were downregulated in the OP group (**Figures 2A, B**). The overlapping of the 1,002 DEGs with 327 LRGs led to the identification of 8 candidate genes (**Figure 2C**). Functional enrichment analysis demonstrated that the eight candidate genes were enriched in 13 GO terms (*P* < 0.05) (**Supplementary Table S4**), including rRNA binding (**Figure 2D**), and 5 pathways (*P* < 0.05) (**Supplementary Table S5**), including the spliceosome (**Figure 2E**). These results suggest that the candidate genes were associated with ribosome biogenesis. The PPI network revealed that elongation of mitochondrial genome 1 (EMG1), ribosomal RNA processing protein 1 homolog B (RRP1B), and far upstream element-binding protein 1 (FUBP1) were highly correlated with other genes (confidence > 0.4) (**Figure 2F**).

### 3.2 CSRP2 and FUBP1 are biomarkers for predicting osteoporosis risk ​in individuals with low BMD/PBM

**Figure 3:**
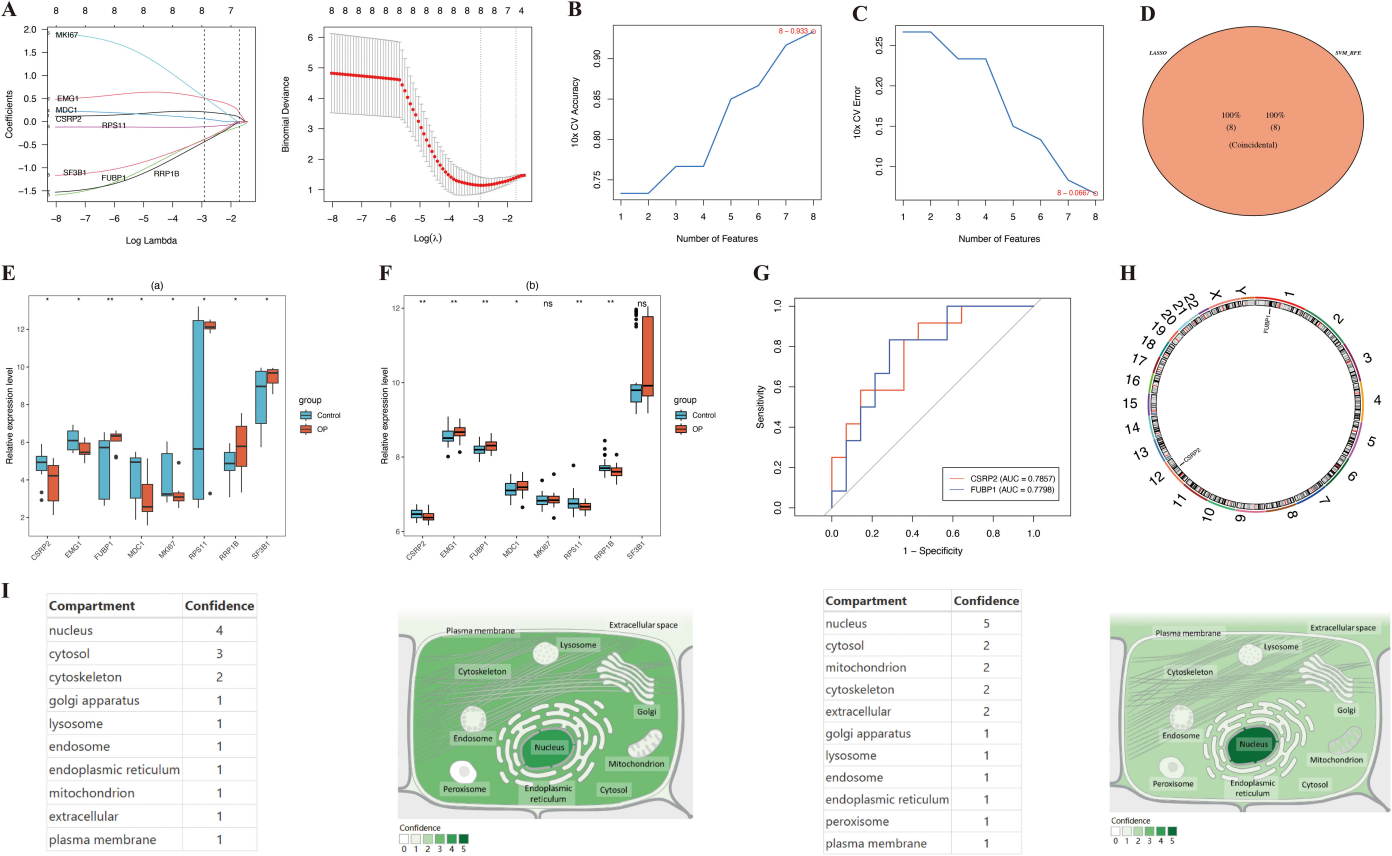
Identification of lactylation-related biomarkers in osteoporosis. **(A-C) **LASSO regression and SVM-RFE algorithms identified key candidate genes. **(D)** Intersection analysis revealed eight core lactylation-related genes. **(E, F)** Multi-cohort validation confirmed CSRP2 downregulation and FUBP1 upregulation in low PBM/BMD group. **(G)** ROC curves demonstrated diagnostic potential of both biomarkers. **(H, I)** Genomic and subcellular localization highlighted nuclear enrichment. *p < 0.05, **p < 0.01, ns, not significant.

LASSO genes were identified from the LASSO analysis, namely *EMG1* (Q16527), *FUBP1* (Q96AE4), mediator of DNA damage checkpoint protein 1 (*MDC1*, Q14676), proliferation marker protein Ki-67 (*MKI67*, P46013), small ribosomal subunit protein uS17 (*RPS11*, P62280), *RRP1B* (Q91YK2), and splicing factor 3B subunit 1 (*SF3B1*, O75533) (**Figure 3A**). Similarly, eight SVM-RFE genes were identified from the SVM-RFE analysis, including *RPS11*, *RRP1B*, *EMG1*, *MDC1*, cysteine and glycine-rich protein 2 (*CSRP2, Q16527*), *SF3B1*, *FUBP1*, and *MKI67* (**Figures 3B, C**). The overlapping of the LASSO and SVM-RFE genes led to the identification of eight feature genes, namely *RPS11*, *RRP1B*, *EMG1*, *MDC1*, *CSRP2*, *SF3B1*, *FUBP1*, and *MKI67* (**Figure 3D**). Expression analysis indicated that *CSRP2* and *FUBP1* showed significant variation in expression in the GSE7158 and GSE56815 datasets (*P* < 0.05), with *CSRP2* being significantly downregulated and *FUBP1* being significantly upregulated in the low PBM/BMD group. However, *MDC1*, *EMG1*, *RRP1B*, and *RPS11* showed opposing expression trends in the two datasets. Meanwhile, *SF3B1* and *MKI67* showed no significant variations in expression in the GSE56815 dataset (*P* > 0.05) (**Figures 3E, F**). Therefore, *CSRP2* and *FUBP1* were identified as the candidate biomarkers for OP. The AUC values of *CSRP2* and *FUBP1* in the GSE7158 dataset were 0.7857 and 0.7798, respectively (**Figure 3G**), indicating that they exhibited good diagnostic capabilities for OP. Subsequently, correlation analysis of *CSRP2/FUBP1* expression and PBM/BMD revealed that *CSRP2* expression was significantly positively correlated with PBM (r = 0.494, *P* = 0.010) and BMD (r = 0.318, *P* = 0.004), while *FUBP1* expression was significantly negatively correlated with PBM (r = −0.483, *P* = 0.012) and BMD (r = −0.293, *P* = 0.008). These findings suggest that both *CSRP2* and *FUBP1* may be involved in OP pathogenesis by regulating bone mass accumulation (**Supplementary Figure S1**). Chromosomal localization analysis revealed that the *CSRP2* and *FUBP1* genes were located on human chromosomes 12 and 1, respectively (**Figure 3H**), while subcellular localization revealed that *CSRP2* and *FUBP1* were mainly expressed in the nucleus (**Figure 3I**). These findings lay a foundation for understanding the roles of CSRP2 and FUBP1 in various biological processes in OP.

### 3.3 The nomogram of biomarkers was constructed and evaluated

**Figure 4:**
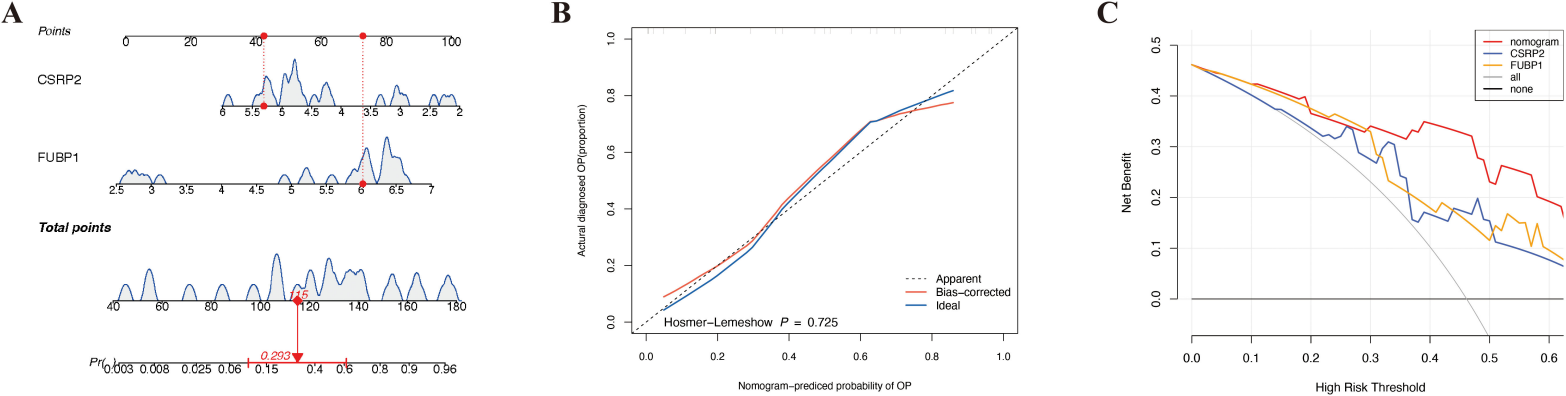
Development and validation of a lactylation-driven nomogram for osteoporosis risk stratification. **(A)** Nomogram integrating CSRP2 and FUBP1 expression levels to predict OP occurrence. **(B)** Calibration curve demonstrating strong concordance between predicted (x-axis) and observed (y-axis) OP probabilities. **(C)** Decision curve analysis showing superior net clinical benefit of the nomogram over single-biomarker models and extreme strategies across threshold probabilities.

A nomogram for predicting OP onset was created based on the selected biomarkers (**Figure 4A**). In the calibration curve, the *P* value of the HL test was 0.725 (**Figure 4B**). DCA revealed that the net utility of the model exceeded that of any single factor (**Figure 4C**), demonstrating that the nomogram exhibited high predictive ability for OP onset.

### 3.4 CSRP2 and FUBP1 regulate OP pathogenesis through immune-related pathways

**Figure 5:**
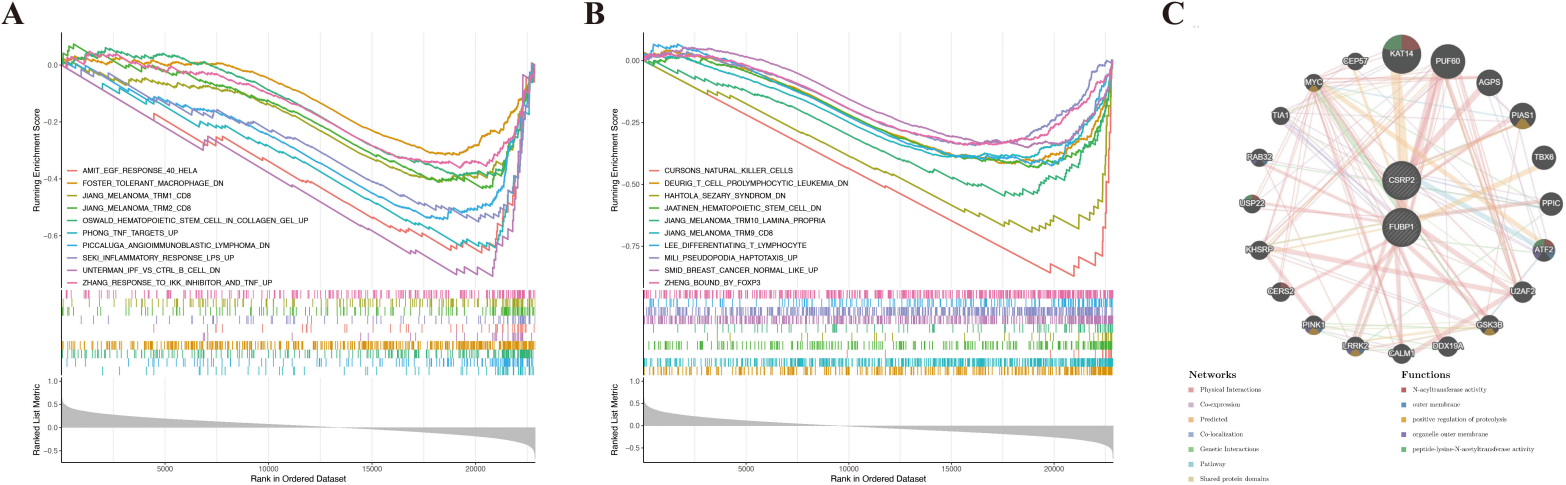
Functional enrichment and interaction networks of lactylation-related biomarkers in osteoporosis. **(A, B)** Gene set enrichment analysis of CSRP2 **(A)** and FUBP1 **(B)** in osteoporosis. **(C)** Gene-gene interaction network of CSRP2/FUBP1 and their top 20 functional partners.

GSEA showed that *CSRP2* and *FUBP1* were significantly enriched in 118 and 72 pathways, respectively (|NES| > 1, Padj < 0.05) (**Supplementary Table S6**). Notably, *CSRP2* was enriched in impaired M2 macrophage polarization (foster tolerant macrophage DN), tumor necrosis factor (TNF)-α hyperactivation (Phong TNF targets Up), and Toll-like receptor-4 (TLR4)-mediated inflammation (Seki inflammatory response LPS Up), while *FUBP1* was enriched in natural killer (NK) cell activation (Cursons’ NK cells), hematopoietic stem cell (HSC) dysfunction (Jaatinen HSC DN), and T cell differentiation (Lee differentiating T lymphocyte) (**Figures 5A, B**). These findings suggest that *CSRP2* and *FUBP1* are closely associated with immune cell regulation and mechanistically link *CSRP2* to pro-inflammatory bone loss and *FUBP1* to lymphocyte-mediated imbalance in bone remodeling. The constructed GGI network further identified the functional partners (e.g., KAT14) of *CSRP2* and *FUBP1*, which were involved in N-acyltransferase activity and proteolysis regulation (**Figure 5C**).

### 3.5 FUBP1 was significantly linked with MSCs

**Figure 6:**
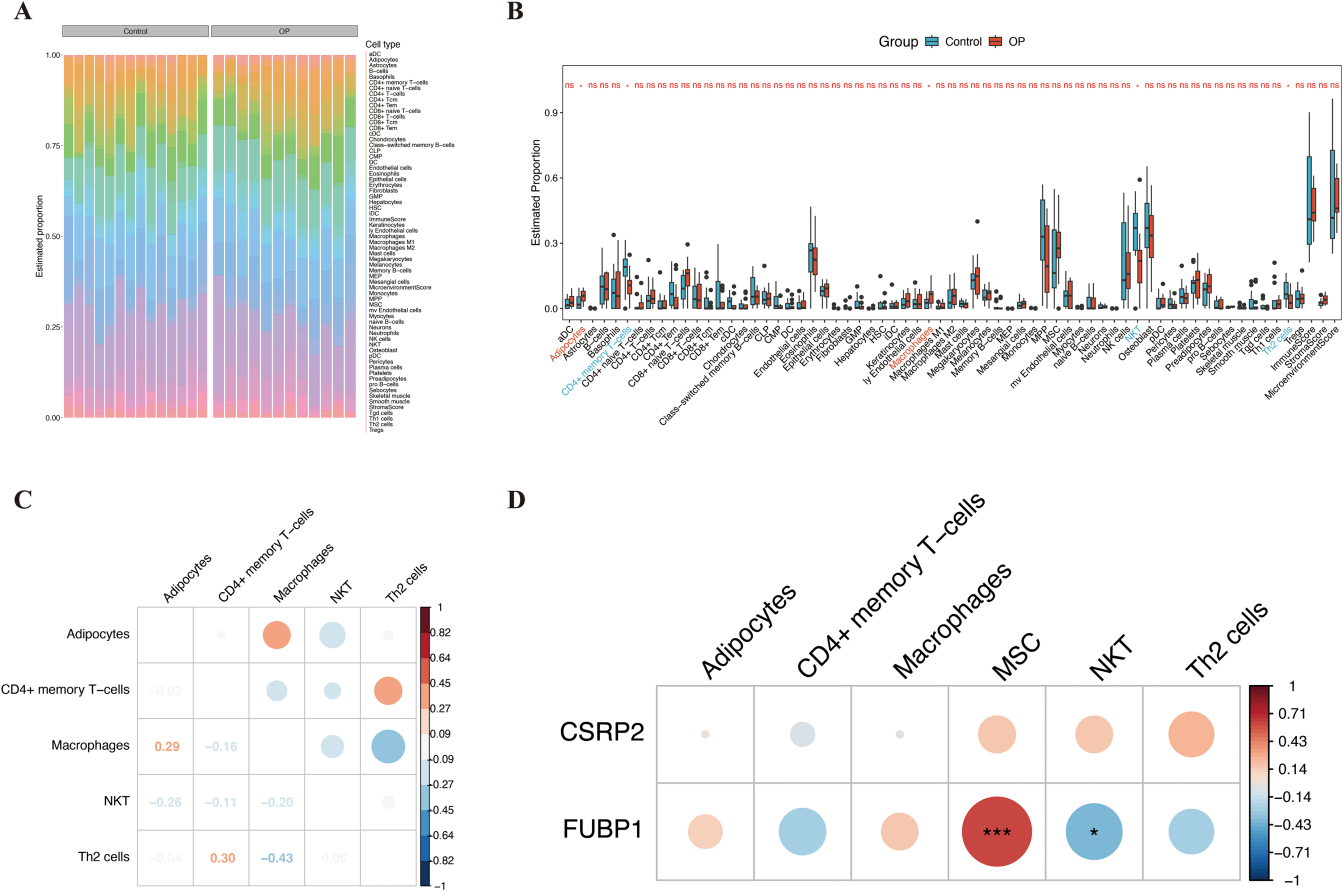
Immune infiltration landscape and correlation of key biomarkers in osteoporosis. **(A)** Heatmap depicting infiltration levels of 64 immune cell types across OP and control groups. **(B)** Differential immune cell infiltration between groups, highlighting adipocytes, CD4+ memory T cells, macrophages, NKT cells, and Th2 cells. **(C)** Spearman correlation network of differentially infiltrated immune cells, with edge thickness proportional to correlation strength. **(D)** FUBP1 exhibited the strongest positive correlation with mesenchymal stem cells and negative correlation with NKT cells. Analyses were performed using the xCell algorithm. *p < 0.05, ***p < 0.001, ns, not significant.

Immune cell infiltration analysis of the 64 types of immune cells revealed that the OP and control groups showed significant variation in the infiltration of adipocytes, CD4+ memory T cells, macrophages, NK T cells (NKT cells), and Th2 cells (*P* < 0.05) (**Figures 6A, B**). Correlation analysis of the differentially infiltrated immune cells indicated that CD4+ memory T cells were significantly positively correlated to Th2 cells (cor = 0.30), while Th2 cells were significantly negatively correlated to macrophages (cor = −0.43) (**Figure 6C**). *FUBP1* was significantly positively correlated with MSCs (cor > 0.61, *P* < 0.001) and significantly negatively correlated with NKT cells (cor < −0.40, *P* < 0.05) (**Figure 6D**; **Supplementary Table S7**).

### 3.6 The regulatory network of biomarkers was constructed

**Figure 7:**
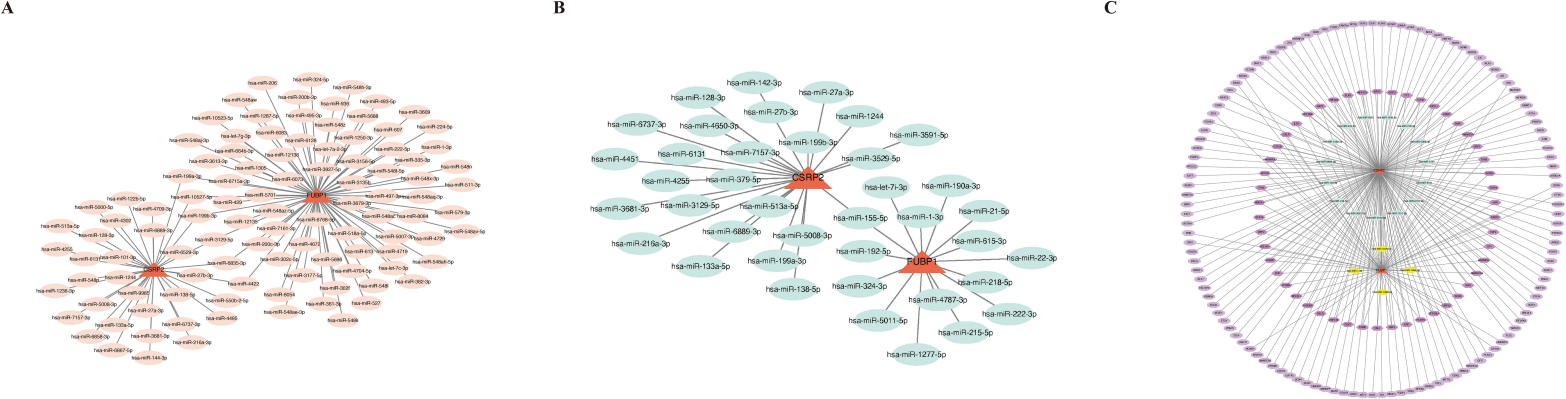
Regulatory networks of lactylation-related biomarkers in osteoporosis. **(A)** miRNA-mRNA interaction network for CSRP2 and FUBP1 predicted by the miRDB database. **(B)** miRNA-mRNA interaction network from the miRTarBase database. **(C)** Integrated TF-mRNA-miRNA regulatory network, showing transcription factors (TFs, pink nodes) and shared miRNAs (blue nodes) co-targeting CSRP2 and FUBP1.

In the miRDB database, 35 and 77 miRNAs were found to target *CSRP2* and *FUBP1*, respectively, while in the miRTarBase database, 25 and 15 miRNAs were found to target *CSRP2* and *FUBP1*, respectively. Based on these results, miRNA–mRNA networks were constructed for the miRDB and miRTarBase databases (**Figures 7A, B**). Overlapping of miRNAs from the two databases led to the identification of 19 intersection miRNAs (**Supplementary Table S8**). In the KnockTF database, 200 and 73 TFs were predicted to target *CSRP2* and *FUBP1*, respectively (**Supplementary Table S9**). Based on these results, a TF–mRNA–miRNA network was then constructed (**Figure 7C**). Further analysis revealed that multiple miRNAs (hsa-miR-27b-3p, hsa-miR-133a-5p, and hsa-miR-6131) and TFs (FOXM1, TAL1, JUN, MAF, and NFATC3) could target both *CSRP2* and *FUBP1* simultaneously.

### 3.7 Drug prediction and molecular docking were performed for biomarkers

**Figure 8:**
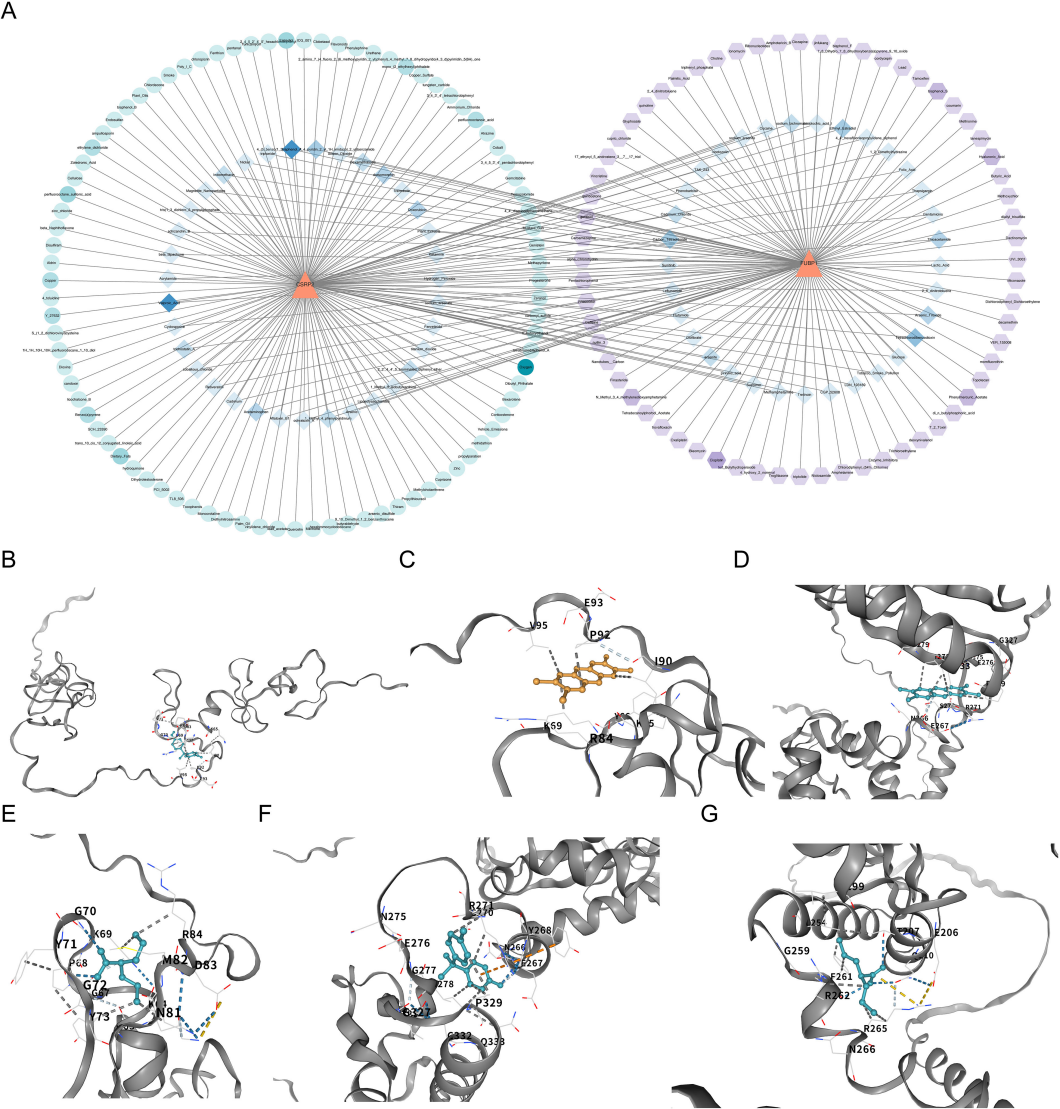
Drug-biomarker interactions and molecular docking validation of lactylation-related biomarkers in osteoporosis. **(A)** Network analysis of potential drugs targeting CSRP2 and FUBP1, constructed using the Comparative Toxicogenomics Database and visualized with Cytoscape. **(B-D)** Molecular docking of CSRP2 with BPA, TCDD, and VPA. **(E-G)** Molecular docking of FUBP1 with TCDD, BPA and VPA.

In the CTD database, 155 and 132 drugs were predicted to target CSRP2 (Protein template PDB: 1IBI) and FUBP1 (Protein template PDB: 6Y24), respectively (**Supplementary Tables S10**, **S11**). Based on these results, a drug–biomarker network was constructed (**Figure 8A**). The top three drugs with the highest binding ability for CSRP2 were bisphenol A (BPA, CAS: 80-09-1, and ΔGbind: -6.1 kcal/mol), tetrachlorodibenzodioxin (TCDD, CAS: 1746-01-6, and ΔGbind: -5.7 kcal/mol) and valproic acid (VPA, CAS: 1069-66–5 and ΔGbind: -5.0 kcal/mol) (**Figures 8B–D**), while the top three drugs with the highest binding ability for FUBP1 were TCDD (ΔGbind: -7.1 kcal/mol), BPA (ΔGbind: -6.8 kcal/mol), and VPA (ΔGbind: -4.6 kcal/mol) (**Figures 8E–G**). The chemical structural formulas of these compounds are depicted (**Supplementary Figure S2**). These results indicate that BPA and TCDD exhibit strong binding affinity to CSRP2 and FUBP1; however, the clinical application of BPA and TCDD is limited by their toxicity. Therefore, these findings should be regarded as the starting point for target validation rather than a therapeutic recommendation.

### 3.8 Annotation yielded eight cell types

Given the CD271+ cell sorting strategy of the GSE147287 dataset, the annotated cell types represent populations within the mesenchymal lineage niche and do not encompass all immune cells in unselected bone marrow.​​ ​​Of the 2,887 cells and 9,654 genes identified in the GSE147287 dataset, 2,353 and 7,907 were retained after quality control (**Supplementary Figures S3A, B**). The top 2,000 HVGs were then identified (**Supplementary Figure S3C**). After evaluating the cumulative contribution of the PCs to the overall data, 20 PCs were selected for t-SNE clustering (**Supplementary Figures S3D–F**). All high-quality cells were classified into 13 distinct cell clusters (**Supplementary Figure S3G**), of which 8 were annotated as BM-MSCs, neutrophils, monocytes, B cells, T cells, NK cells, nucleated red blood cells, and HSCs (**Supplementary Figure S3H**). The marker genes showed specificity towards different cell clusters (**Supplementary Figure S3I**). ​It should be emphasized that ‘T cells’ and ‘NK cells’ identified herein derive specifically from CD271+-sorted mesenchymal progenitors, representing niche-associated immune-like populations rather than conventional hematopoietic immune cells.

### 3.9 BM-MSCs, T cells, NK cells, and HSCs were identified as key cells

Among the eight annotated cell types, NK cells mainly expressed *CSRP2*, while BM-MSCs, T cells, and HSCs mainly expressed *FUBP1* (**Supplementary Figures S4A, B**). Moreover, the *CSRP2* and *FUBP1* expression levels were significantly correlated with the infiltration abundances of these immune cells (Spearman r > 0.5, *P* < 0.001, **Figure 6D**). Therefore, BM-MSCs, T cells, NK cells, and HSCs were identified as key cells in OP. Further analysis of the distribution of the four key cell types and the expression and distribution of *CSRP2* and *FUBP1* among these cell types (**Supplementary Figures S4C, D**) revealed that the expression of *CSRP2* was the highest in NK cells, and the expression of *FUBP1* was the highest in HSCs (**Supplementary Figure S4E**). Notably, FUBP1 eenrichment in HSCs aligns with the established role of HSC aging in bone loss pathogenesis (44). The cell–cell communication network showed that among the key cells, BM-MSCs had a relatively large number of interactions with NK cells, with relatively high interaction intensity (**Supplementary Figure S4F**). The receptor–ligand pairs were then identified for all the annotated cell types (**Supplementary Figure S4G**). For example, the interactions between BM-MSCs and neutrophils were mainly mediated by CXCL12–CXCR4 and RETN–CAP1.

### 3.10 Differentiation states of key cells and expression changes of biomarkers were explored

Following secondary dimensionality reduction clustering, BM-MSCs, T cells, NK cells, and HSCs clustered into 5, 3, 2, and 2 cell subtypes, respectively (**Supplementary Figures S5A–D**). According to the differentiation time, cells of different subtypes were arranged along the developmental trajectory, with a darker blue color indicating earlier cell differentiation. BM-MSCs had 4 differentiation states, and BM-MSC subtypes 0, 1, and 3 differentiated earlier (**Supplementary Figure S6A**), while T cells had 8 differentiation states, and T cell subtype 2 differentiated the earliest (**Supplementary Figure S6B**). The expression of key genes was relatively conserved during BM-MSC and T cell differentiation (**Supplementary Figures S6C, D**). NK cells had 7 differentiation states, and NK cell subtype 0 differentiated earlier (**Supplementary Figure 7A**). During NK cell differentiation, *CSRP2* expression first increased and then decreased, while *FUBP1* expression remained conserved (**Supplementary Figure S7B**). HSCs had 7 differentiation states, and HSC subtype 1 differentiated earlier (**Supplementary Figure S7C**). During HSC differentiation, *FUBP1* expression slowly increased and then decreased slightly, while *CSRP2* expression remained conserved (**Supplementary Figure S7D**).

### 3.11 The expression of biomarkers was verified

**Figure 9:**
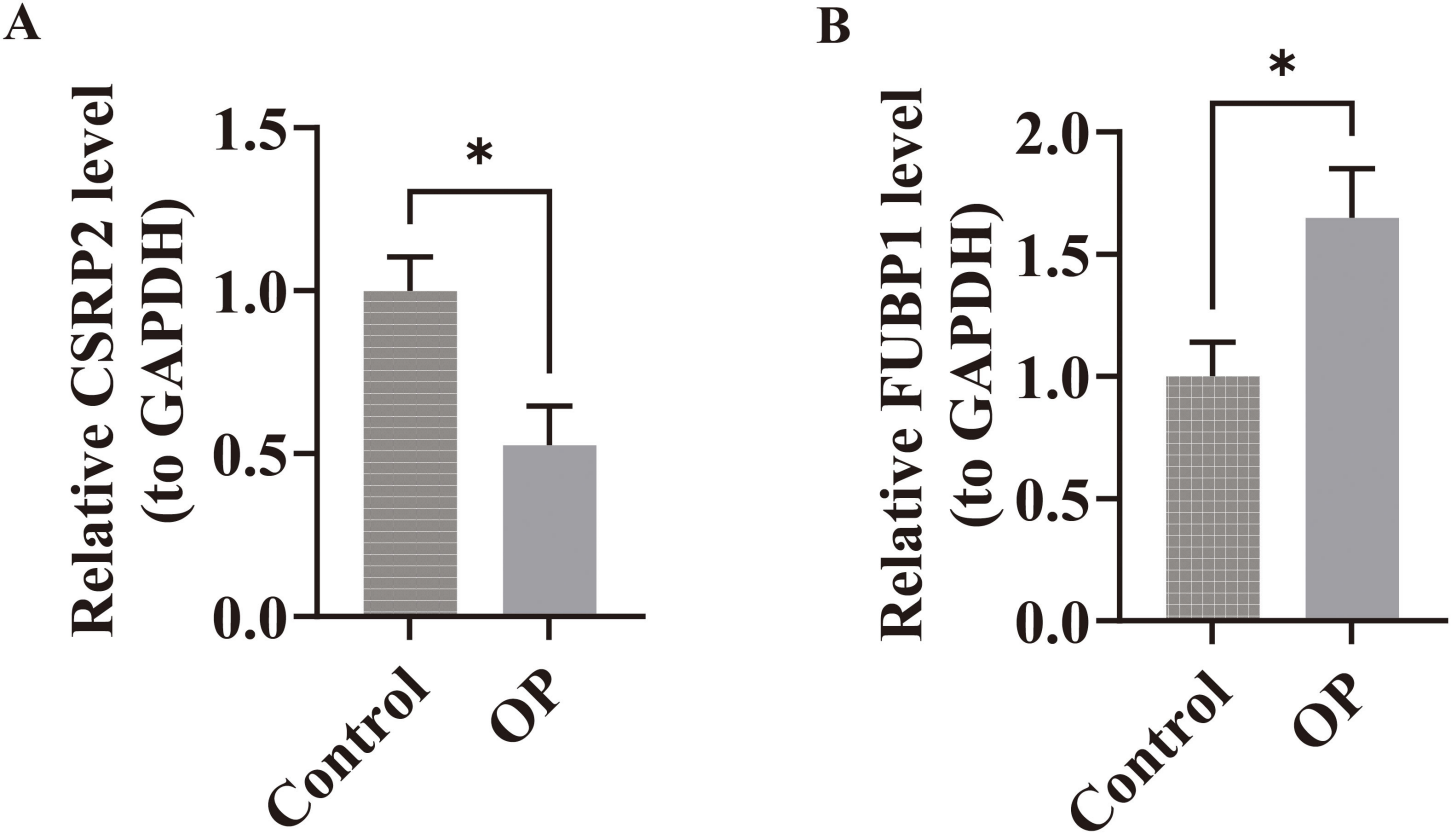
Experimental validation of biomarker expression in osteoporosis patients. **(A)** RT-qPCR analysis revealed significant downregulation of CSRP2 mRNA levels in peripheral blood mononuclear cells from OP patients compared to controls. **(B)** Conversely, FUBP1 expression was markedly upregulated in OP patients. *p < 0.05.

Within the GSE7158 and GSE56815 datasets, the expression of *CSRP2* was downregulated and *FUBP1* was upregulated in the OP groups (*P* < 0.05) (**Figures 3F, G**). RT-qPCR analysis further revealed that *CSRP2* expression was downregulated and *FUBP1* expression was upregulated in clinical OP samples (*P* < 0.05) (**Figures 9A, B**), consistent with the above findings, thus verifying the accuracy of bioinformatics analysis.

## 4 Discussion

Lactylation enhances bone formation by upregulating the expression of key osteogenic genes, thereby alleviating OP symptoms through improved bone remodeling (45). This study identified two lactylation-associated biomarkers, CSRP2 and FUBP1, by analyzing the transcriptomic data of OP patients from publicly available datasets. Subsequently, bioinformatics approaches were employed to investigate the biological pathways associated with these biomarkers in OP pathogenesis and analyze their expression profiles and spatial distribution within key cell populations using single-cell datasets, thereby establishing novel directions for exploring the underlying mechanisms and therapeutic targets in OP.

*CSRP2* expression was significantly downregulated and *FUBP1* expression was significantly upregulated in the OP groups of the GSE7158 and GSE56815 datasets. These results were further validated through RT-qPCR of clinical OP samples. The *CSRP2* gene encodes CRP2, which contains two LIM domains and regulates cellular differentiation and proliferation to regulate growth (46). Muscle LIM Protein (MLP) is a LIM-only protein that is specifically expressed in striated muscle cells, and it regulates the osteocytic cytoskeleton and mechanostress signaling transduction (47). As CSRP2 and MLP both contain LIM domains, which are often associated with mechanoregulatory functions, we propose that CSRP2 may also play a role in mechanoregulation, although its specific functions may differ from those of MLP (48). Considering the high mechanosensitivity of osteocytes, pathological downregulation of CSRP2 may impair osteocytic mechanosensing and signaling transduction, leading to disrupted bone remodeling and increased OP risk. Mechanical stress generated by muscle contraction is a critical determinant of bone density maintenance. Mulim et al. (49) demonstrated that CSRP2 plays a key role in muscle development. Thus, marked downregulation of CSRP2 may compromise muscle function, thereby diminishing skeletal loading stimuli and elevating OP risk via the muscle–bone crosstalk axis (50). Parathyroid hormone-related peptide (PTHrP) inhibits hypertrophic chondrocyte differentiation by activating the PTH1R receptor, thereby delaying the closure of the epiphyseal growth plates (51). As a target gene of PTHrP, *CSRP2* may indirectly influence bone formation and resorption homeostasis by modulating chondrocyte differentiation, which is critical for endochondral ossification.

FUBP1 is a multifunctional nucleic acid-binding protein that plays a central role in cell proliferation, differentiation, and survival by regulating transcription, RNA metabolism, and viral replication (52). Transforming growth factor-beta (TGF-β) serves as a critical regulator of bone metabolism, and FUBP1 may disrupt bone formation–resorption coupling by overactivating the TGF-β/Smad signaling pathway, thereby leading to reduced bone mass and deterioration of bone microstructure (52, 53). V-myc myelocytomatosis viral oncogene homolog (MYC) is a TF and oncoprotein that regulates gene transcription by interacting with multiple proteins, and FUBP1 serves as a critical activator of *MYC* transcription (54, 55). MYC maintains the proliferative capacity of osteoprogenitor cells by activating cell cycle-related genes; however, elevated MYC expression may antagonize the activity of key osteogenic differentiation TFs, such as RUNX2, thereby delaying terminal differentiation (56, 57). In this study, the RT-qPCR results demonstrated that *FUBP1* expression was upregulated in OP patients, suggesting its potential as a key mediator in OP pathogenesis. Mechanistically, FUBP1 may regulate bone remodeling by modulating both the TGF-β/Smad and MYC signaling pathways.

In this study, an OP risk prediction nomogram was developed based on *CSRP2* and *FUBP1* expression, and its performance was validated through calibration curves, HL test, and DCA. The calibration curve demonstrated that the nomogram’s predicted probabilities aligned closely with the observed probabilities (slope close to 1). The HL test yielded a *P* value of 0.725 (*P* > 0.05), indicating excellent model calibration without significant deviation (58). Lastly, DCA revealed that the nomogram achieved a significantly higher net benefit within the threshold probability range of 0.3–0.6 compared to extreme strategies and the single-gene model, thereby demonstrating its clinical utility (59).

This study utilized GSEA to identify potential CSRP2- and FUBP1-regulated pathways in the OP bone microenvironment. Among the top 10 enriched gene sets, *CSRP2* exhibited significant enrichment in three inflammatory response-related modules: foster tolerant macrophage DN, Phong TNF targets Up, and Seki inflammatory response LPS Up. Tolerant macrophages, primarily classified as M2 macrophages, are essential for maintaining bone homeostasis, and their downregulation promotes a chronic pro-inflammatory microenvironment dominated by cytokines, which disrupts bone metabolic homeostasis, suppresses osteoblast function, and enhances osteoclast activity (59, 60). Mechanistic studies reveal that TNF-α stimulates stromal cells and osteoblasts to express receptor activator of nuclear factor (NF)-κB (RANK) ligand (RANKL) and macrophage colony-stimulating factor, thereby amplifying osteoclast precursor differentiation via the RANKL/RANK axis (61). Furthermore, elevated TNF-α concentrations suppress osteoblast function through coordinated downregulation of IGF-1 and RUNX2 expression (61). Lipopolysaccharide (LPS), a complex comprising lipid and polysaccharide moieties, activates TLR4-mediated inflammatory cascades in macrophages and monocytes, inducing proinflammatory cytokines and RANKL to exacerbate osteoclastic bone resorption and reduce bone density (62). Consequently, the downregulation of CSRP2 may contribute to OP pathogenesis by disrupting macrophage polarization homeostasis and exacerbating inflammatory signaling pathways that promote bone resorption and inhibit bone formation.

*FUBP1* exhibited significant enrichment in the following gene sets from GSEA: Cursons’ NK cells, Jaatinen HSC DN, and Lee differentiating T lymphocyte. NK cells mediate interleukin (IL)-15-dependent suppression of osteoclastic bone resorption through apoptosis induction; therefore, their functional impairment or homeostatic dysregulation can be a potential mechanistic contributor to OP pathogenesis (63). HSCs, osteoblasts, and osteoclasts coexist within the bone marrow niche. HSC-derived extracellular vesicles (EVs) harbor signaling molecules essential for maintaining marrow homeostasis, and functional suppression of HSCs reduces EV secretion, thereby impairing the osteogenic differentiation capacity of MSCs and diminishing bone formation (64). T lymphocytes, primarily responsible for mediating cellular immune responses, may exacerbate OP through pathological mechanisms. For instance, aberrant secretion of proinflammatory cytokines by dysregulated T cells can stimulate RANKL production in osteoblasts, synoviocytes, and other mesenchymal lineage cells, thereby activating osteoclast differentiation cascades (65). Therefore, FUBP1 may contribute to OP pathogenesis by influencing NK cells, HSCs, and T cells.

Immune cell infiltration analysis revealed that *FUBP1* expression was significantly positively correlated with MSC infiltration and significantly negatively correlated with NKT cells. BM-MSCs, a subset of MSCs, differentiate into osteoblasts and adipocytes. Li et al. demonstrated that aging drives BM-MSCs to preferentially differentiate into adipocytes over osteoblasts, which manifests as increased adipocyte accumulation and decreased osteoblast levels within the marrow cavity (66). Bone marrow adipocytes secrete proinflammatory cytokines, including IL-6 and TNF-α, which activate osteoclastogenesis and suppress osteoblast function, thereby exacerbating the imbalance between bone resorption and formation (66, 67). NKT cells, a subset of T lymphocytes, exhibit dual regulatory effects on bone metabolism. Type I NKT cells promote osteoclast differentiation through CD1d-restricted lipid antigen recognition and subsequent proinflammatory cytokine release (interferon [IFN]-γ/TNF-α) (68–70), whereas type II NKT cells suppress osteoclastogenesis via anti-inflammatory IL-10 secretion, which inhibits RANKL signaling (71). Thus, selective activation of type II NKT cells or inhibition of proinflammatory type I NKT cells may restore bone metabolic homeostasis.

To delineate the pathogenic mechanisms underlying OP, we systematically investigated the regulatory interplay between TFs, miRNAs, and the lactylation-regulated *CSRP2* and *FUBP1* genes. Hsa-miR-133a-5p, a member of the miR-133 family, is implicated in skeletal metabolism regulation (72). MiR-133a is upregulated in OP patients, and it suppresses bone formation by inhibiting Runx2, a key osteogenic TF, thereby blocking bone morphogenetic protein 2-mediated osteogenic differentiation (73). Furthermore, miR-133a promotes MSC differentiation to adipocytes over osteoblasts by upregulating adipogenesis-associated proteins (73). Mechanistic studies found that miR-27a enhances osteogenic differentiation and suppresses osteoclastogenesis by targeting Dickkopf-2 to activate the Wnt/β-catenin signaling pathway, thereby ameliorating bone metabolic imbalance (74). As miR-27b-3p belongs to the miR-27 family, it may share functional similarities. Furthermore, Sun et al. revealed that Jumonji domain-containing protein 3 (JMJD3) cooperates with NF-κB to drive proinflammatory gene expression. Notably, miR-27b exerts dual suppression on both JMJD3 and NF-κB, thereby inhibiting inflammation-associated bone loss (75). JUN, a core component of the AP-1 TF family, significantly upregulates *RUNX2* gene expression in bone- and stromal-progenitor cells, driving osteoprogenitor commitment toward osteoblast lineage and increasing osteoblast numbers (76). However, as a downstream effector of the JNK signaling pathway, JUN accelerates bone resorption by promoting transcription of inflammatory cytokines (IL-6 and TNF-α), thereby establishing a chronic inflammatory microenvironment (77). These findings suggest that JUN exerts cell-type- and disease-stage-dependent biphasic effects, warranting further investigation into its context-specific roles.

In this study, pharmacoinformatic prediction and molecular docking analyses were used to identify high-potency binding interactions between BPA/TCDD and the lactylation-modified targets CSRP2/FUBP1 (**Table 1**). BPA and TCDD were selected as probe molecules due to their established roles in bone metabolism disruption. BPA mimics estrogenic activity to dysregulate bone remodeling via ERα/β interference and Wnt/β-catenin suppression (78–80), while TCDD activates AHR–RANKL signaling to accelerate osteoclastogenesis (81). The observed binding affinity between BPA/TCDD and CSRP2/FUBP1 suggests their potential to modulate CSRP2/FUBP1 activity within bone metabolic networks. CSRP2 serves as a mechanosensitive regulator whose downregulation impairs osteocyte signaling, whereas *FUBP1* overexpression drives inflammatory bone loss via the TGF-β/Smad and MYC pathways. Therefore, targeting these nodes may mitigate lactylation-driven imbalances in bone formation–resorption coupling, which is a core OP pathomechanism. However, the toxicity of BPA and TCDD limits their direct clinical application. Consequently, the findings of this study should be regarded as preliminary evidence for target validation, providing a structural template for developing safer analogues targeting CSRP2/FUBP1, rather than a therapeutic recommendation. Future efforts necessitate screening for specific small-molecule modulators to develop safe therapeutic agents targeting CSRP2 and FUBP1.

**Table 1:** The binding energies of drug-biomarkers.

Energy components(Kcal/mol)		
Target	Compounds	ΔGbind
CSRP2	Bisphenol A Tetrachlorodibenzodioxin Valproic Acid	-6.1 -5.7 -5.0
FUBP1	Bisphenol A Tetrachlorodibenzodioxin Valproic Acid	-6.8 -7.1 -4.6

Single-cell sequencing resolves cellular heterogeneity through single-cell resolution and multi-omics integration, overcoming the averaging bias of bulk sequencing to map cell–cell interactions and differentiation trajectories, thereby enabling high-dimensional deconvolution of complex biological systems (82). Within the CD271+-sorted mesenchymal niche, *CSRP2* expression was significantly high in NK cells, while *FUBP1* expression was significantly high in BM-MSCs, T lymphocytes, and HSCs, suggesting that these immune cells were involved in OP pathogenesis. Studies indicate that NK cells drive OP pathogenesis by secreting IFN-γ and TNF-α, which stimulate osteoclast precursor differentiation and survival (68, 70, 83–85). Notably, this pathogenicity is amplified in the CSRP2-high NK subpopulation identified by single-cell analysis, where cytokine-driven *CSRP2* overexpression activates a p38 MAPK-mediated feedback loop that perpetuates IFN-γ/TNF-α hypersecretion and accelerates trabecular bone loss (68, 86). BM-MSCs are multipotent stem cells residing in the bone marrow niche, and they can differentiate into multiple cell lineages. Impaired osteogenic differentiation potential and enhanced adipogenic propensity of BM-MSCs can lead to OP progression (83), and this imbalance may be driven by oxidative stress-induced DNA damage accumulation (86, 87). FUBP1 dysfunction under oxidative stress impairs DNA repair capacity, which can lead to elevated γH2AX levels and downregulated DNA repair pathways, thereby accelerating cellular senescence and promoting BM-MSC differentiation toward adipogenesis (88). T lymphocytes contribute to glucocorticoid-mediated pathological bone loss by upregulating RANKL, which drives osteoclast differentiation from macrophage lineage precursors, thereby exacerbating bone resorption and structural deterioration (89). As FUBP1 amplifies TGF-β/Smad signaling, which directly activates RANKL expression in T cells via Smad3–TRAF6 complex formation (90), we propose that *FUBP1* overexpression in T cells potentiates RANKL-mediated osteoclastogenesis, ultimately contributing to OP progression. HSCs predominantly reside in the bone marrow and peripheral blood, and HSC aging upregulates Wnt5a to drive myeloid bias and functional decline, which promotes osteoclastogenesis and bone resorption (44). *FUBP1* overexpression may accelerate this aging process by disrupting HSC homeostasis. FUBP1 upregulation or overexpression can trigger p53 pathway dysregulation. FUBP1 loss directly activates p53/p21-mediated senescence, while its persistent overexpression can disrupt RNA splicing fidelity, leading to genomic instability and p53 hyperactivation, thereby promoting HSC aging (52). The p53/p21-mediated senescence cascade promotes myeloid skewing and functional decline, which can lead to osteoclast hyperactivity and bone loss. Therefore, *FUBP1* overexpression likely exacerbates OP by amplifying Wnt5a-mediated myeloid bias and osteoclastogenesis in aged HSCs. In this study, pseudotime analysis revealed that *CSRP2* exhibits nonlinear expression dynamics in NK cells, which may explain its dual regulatory role in the bone immune microenvironment. The cell-specific expression patterns of *CSRP2* and *FUBP1* suggest their potential utility as dual biomarkers for OP diagnosis and progression evaluation. Expression profiling of these regulators may provide a molecular basis for risk prediction, disease staging, and treatment efficacy assessment in the clinical management of OP.

Altogether, our findings indicate that CSRP2 is a critical regulator of bone’s mechanosensory and inflammatory systems and FUBP1 is a key regulator of osteoblast differentiation and immune cell function. The downregulation of *CSRP2* can impair osteocyte signaling and disrupt the vital muscle–bone crosstalk, contributing to diminished bone formation and strength. In contrast, the upregulation of *FUBP1* can disrupt the TGF-β/Smad signaling pathway and hyperactivate MYC-driven proliferation, thereby lowering osteogenic commitment and leading to excessive bone resorption and compromised bone mass. This dual dysregulation by CSRP2 and FUBP1 provides the mechanistic framework for understanding the role of lactylation in OP progression. Future studies are warranted to delineate the specific molecular mechanisms by which lactylation modifies CSRP2 and FUBP1 activity and contributes to OP pathogenesis. Furthermore, while this study primarily identifies CSRP2 and FUBP1 as lactylation-associated biomarkers in OP, their clinical translational value warrants further elucidation through the optimization of therapeutic strategies. Intervention strategies can leverage the characteristic significant downregulation of *CSRP2* in OP to target lactylation modification or inflammatory pathways (e.g., TNF-α). For instance, modulating lactylation levels via histone deacetylase inhibitors or blocking the inflammatory cascade using TNF-α monoclonal antibodies may restore CSRP2-mediated bone remodeling homeostasis. Regarding the aberrant upregulation of FUBP1, intervention strategies can focus on inhibiting the hyperactivation of the TGF-β/Smad or MYC signaling pathways. For instance, TGF-β receptor antagonists or MYC transcriptional inhibitors can be used to disrupt FUBP1-driven osteoclast differentiation and bone resorption processes. Collectively, this study provides a promising novel avenue for precision diagnostics and targeted therapies for OP.

While this study provides novel insights, several limitations should be considered. First, the OP cohorts were defined by low PBM or BMD, which are established precursors to OP rather than formal diagnoses. It is important to emphasize that suboptimal peak bone mass acquisition predisposes individuals to accelerated bone loss later in life, while declining BMD directly reflects the progressive deterioration of bone microarchitecture characteristic of OP pathogenesis. Our observed dysregulation of *CSRP2* and *FUBP1* expression in these high-risk cohorts may represent early molecular events in this continuum. Specifically, CSRP2 deficiency potentially impairs the mechanical adaptation essential for bone mass maintenance, whereas *FUBP1* overexpression likely exacerbates bone resorption processes. Second, although our bioinformatics analyses delineated the potential functional roles and associated pathways for CSRP2 and FUBP1, their sole reliance on transcriptomics data is insufficient. Consequently, there is a need to establish a causal relationship between CSRP2/FUBP1 and OP pathogenesis and decipher the biological significance of post-translational modifications (notably lactylation) based on multi-omics datasets and direct experimental validation. Third, the sample sizes utilized in this study present constraints. The training cohort and our RT-qPCR validation cohort are relatively small, inherently introducing greater variability and reducing statistical power to detect precise effects. Moreover, the scRNA-seq analysis was derived from only one OP patient (GSE147287), severely limiting the generalizability of the observed cellular dynamics due to the inability to account for patient heterogeneity. Fourth, biomarker discovery primarily utilized PBMCs, while validation involved bone marrow cells, warranting potential compartment-specific differences. Consequently, future research should prioritize the inclusion of larger, rigorously phenotyped cohorts and integrate multi-omics data (e.g., proteomics, metabolomics). This expanded dataset will facilitate (1) large-scale bioinformatics analyses to enhance biomarker validation reliability, model stability, and effect size estimation precision, and (2) comprehensive single-cell profiling to resolve cellular heterogeneity within the OP bone marrow niche, thereby addressing the limitations associated with the current sample sizes. Additionally, future studies can employ genetic manipulation techniques (e.g., knockdown or overexpression) to construct cellular and animal models. This systematic investigation aims to elucidate the specific functions and regulatory mechanisms of CSRP2 and FUBP1 in OP, while concurrently validating the PBMC-derived biomarkers both within the experimental models and in independent blood sample datasets to exclude potential compartment-specific effects. Collectively, these integrated approaches are crucial for establishing the biological significance and causal contributions of these biomarkers to OP pathophysiology, paving the way towards identifying potential diagnostic markers and therapeutic strategies for the clinical management of OP.

## 5 Conclusion

Our integrative multi-omics analysis identified CSRP2 and FUBP1 as novel lactylation-modified biomarkers in OP. The downregulation of *CSRP2* and upregulation of *FUBP1* in high-risk and OP-associated contexts were robustly validated across cohorts and experiments, demonstrating their potential as diagnostic indicators, supported by a predictive nomogram model. Functional, single-cell, and pharmacoinformatic investigations revealed crucial roles for these biomarkers in regulating inflammatory responses, immune cell differentiation (particularly within NK cells, BM-MSCs, T cells, and HSCs), and cellular dynamics in the bone microenvironment, while also suggesting potential interactions with environmental factors. Collectively, these findings provide significant new insights into the molecular pathogenesis of OP linked to lactylation metabolism and establish a foundation for the development of targeted diagnostic and therapeutic strategies.

## Data Availability

The datasets presented in this study can be found in online repositories. The names of the repository/repositories and accession number(s) can be found in the article/**Supplementary Material**.

## Supplementary material

Supplementary Figure 1 Correlation analysis between CSRP2/FUBP1 expression and PBMC/BMD. Correlation analysis between CSRP2 expression and PBM **(A)** and BMD **(B)**. Correlation analysis between FUBP1 expression and PBM **(C)** and BMD **(D)**.

Supplementary Figure 2 Chemical structure diagrams of predicted drugs (**(A)** bisphenol **(A, B)** Tetrachlorodibenzodioxin, **(C)** Valproic Acid).

Supplementary Figure 3 Single-cell RNA sequencing quality control and dimensionality reduction analysis.​**(A)** Violin plots displaying raw cellular distributions of total genes, RNA counts, and mitochondrial gene percentage across all cells before quality control. **(B)** Post-filtering distributions showing retained high-quality cells. **(C)** Selection of top 2,000 highly variable genes for downstream analysis. **(D)** PCA plot of the first two principal components, illustrating variance distribution across cells after HVG-based dimensionality reduction. **(E)** Jackstraw analysis confirming significant PCs. **(F)** Elbow plot selecting 20 PCs for t-SNE clustering. **(G)** t-SNE visualization of 13 initial cell clusters. **(H)** ​Annotated cell types based on marker genes. **(I)** Dot plot of cell type-specific marker expression.

Supplementary Figure 4 Single-cell RNA sequencing reveals biomarker expression dynamics and intercellular communication in osteoporosis. **(A, B)** FeaturePlot visualization of CSRP2 and FUBP1 expression across 8 annotated cell types. CSRP2 exhibited predominant expression in NK cells, while FUBP1 was enriched in BM-MSCs, T cells, and HSCs. **(C, D)** t-SNE plots showing spatial distribution of key cell populations and biomarker expression patterns. **(E)** DotPlot quantification confirming elevated CSRP2 in NK cells and FUBP1 in HSCs. **(F)** Cell-cell communication network analyzed by CellChat, highlighting prominent interactions between BM-MSCs and NK cells. **(G)** Receptor-ligand pairs mediating intercellular crosstalk.

Supplementary Figure 5 Secondary dimensionality reduction and clustering of key cell populations in osteoporosis bone marrow. **(A)** BM-MSCs cells subdivided into 5 distinct subtypes via t-SNE clustering. **(B)** T cells segregated into 3 transcriptional subtypes. **(C)** NK cells partitioned into 2 subpopulations. **(D)** HSCs resolved into 2 heterogeneous clusters.

Supplementary Figure 6 Differentiation trajectories and biomarker expression dynamics in BM-MSCs and T cells. **(A)** Pseudotime trajectory analysis of BM-MSCs showing differentiation states. **(B)** T cell pseudotime trajectory with differentiation progression. **(C, D)** Heatmaps depicting conserved expression patterns of CSRP2 and FUBP1 across BM-MSC **(C)** and T cell **(D)** differentiation states.

Supplementary Figure 7 Pseudotime trajectory analysis reveals dynamic biomarker expression during NK cell and HSC differentiation.​**(A)** t-SNE visualization of NK cell differentiation states, with pseudotime trajectory. Subtype 0 represents earlier differentiation stages. **(B)** Expression dynamics of CSRP2 and FUBP1 across NK cell pseudotime. **(C)** t-SNE plot of HSC differentiation states, with pseudotime trajectory. Subtype 1 marks earlier differentiation. **(D)** FUBP1 expression gradually increases during HSC differentiation before slight decline, while CSRP2 remains conserved.

Supplementary Table 1 327 Lactylation-Related Genes (LRGs)​​.

Supplementary Table 2 ​Marker Genes for Annotated Cell Types in scRNA-seq Analysis.

Supplementary Table 3 Primer Sequences for RT-qPCR Validation of Biomarkers and GAPDH​.

Supplementary Table 4 Gene Ontology Enrichment Analysis.

Supplementary Table 5 Kyoto Encyclopedia of Genes and Genomes​ Pathway Enrichment Analysis.

Supplementary Table 6 Gene Set Enrichment Analysis Results for CSRP2-Associated KEGG Pathways​.

Supplementary Table 7 Correlation Analysis Between Biomarkers and Differentially Infiltrated Immune Cells​.

Supplementary Table 8 Validated and Predicted miRNAs Targeting CSRP2 and FUBP1.

Supplementary Table 9 Predicted Transcription Factors Regulating CSRP2 and FUBP1.

Supplementary Table 10 Potential Drugs Targeting CSRP2 Predicted by Comparative Toxicogenomics Database.

Supplementary Table 11 Potential Drugs Targeting FUBP1 Predicted by Comparative Toxicogenomics Database.
