# Supplementary figures and images for: Identification of lactylation-related biomarkers in osteoporosis from transcriptome and single-cell data
Source: Front Endocrinol (Lausanne). 2025 Aug 25;16:1621878. doi: 10.3389/fendo.2025.1621878 (PMC12414738; doi:10.3389/fendo.2025.1621878)

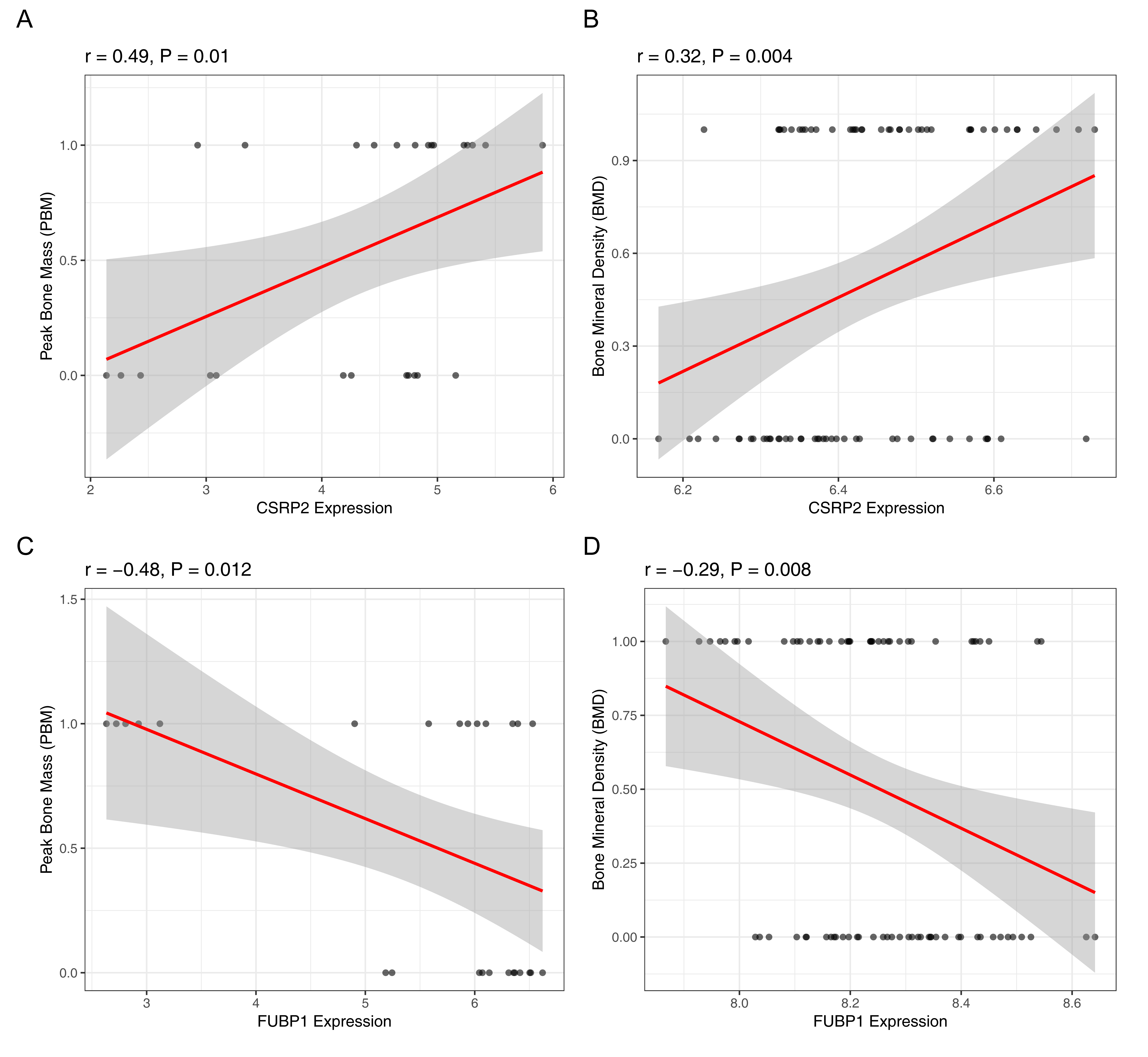

Supplement: Supplementary Figure 1 — Correlation analysis between CSRP2/FUBP1 expression and PBMC/BMD. Correlation analysis between CSRP2 expression and PBM (A) and BMD (B). Correlation analysis between FUBP1 expression and PBM (C) and BMD (D). [file Image1.tif]

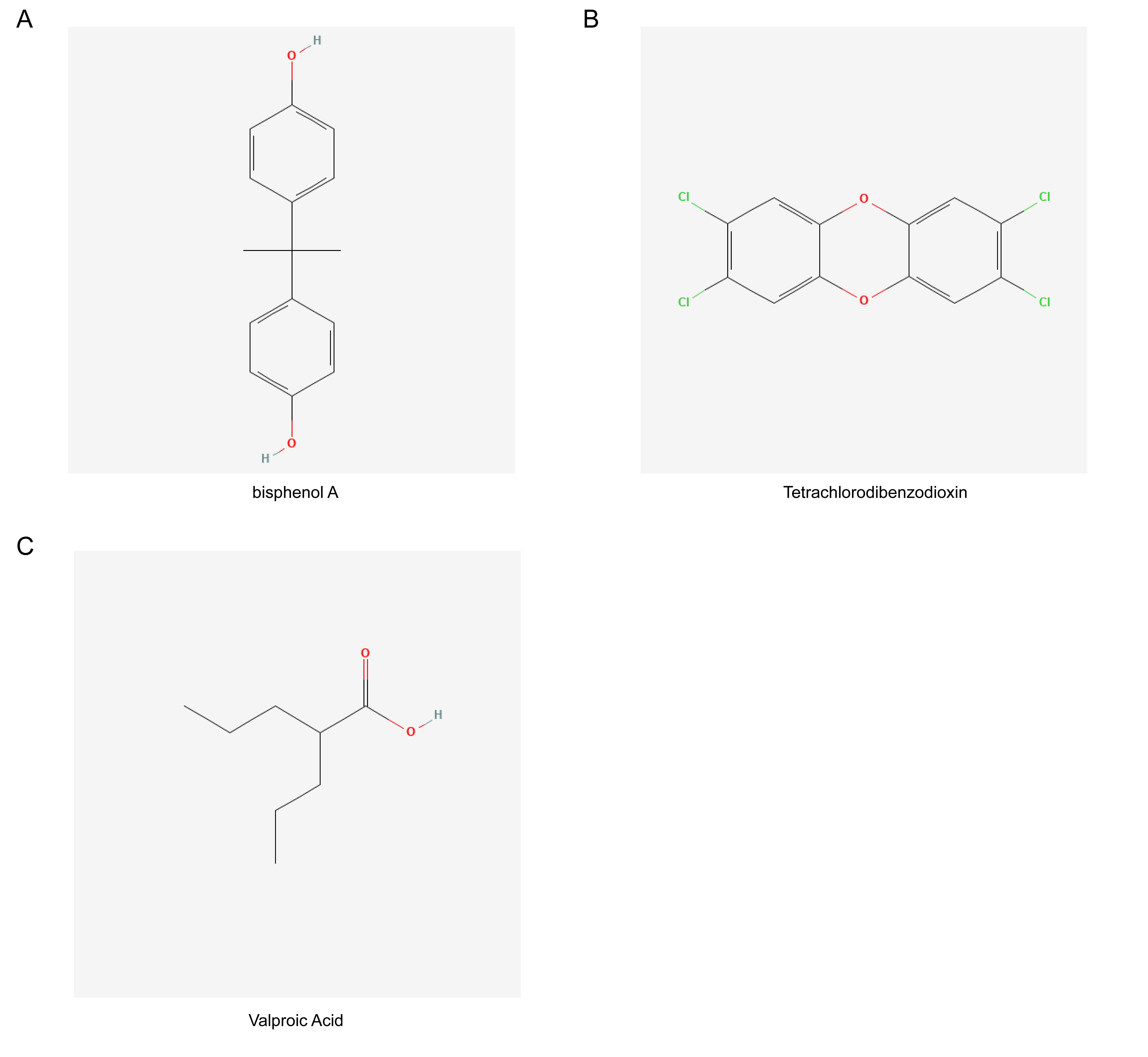

Supplement: Supplementary Figure 2 — Chemical structure diagrams of predicted drugs ((A) bisphenol (A, B) Tetrachlorodibenzodioxin, (C) Valproic Acid). [file Image2.tif]

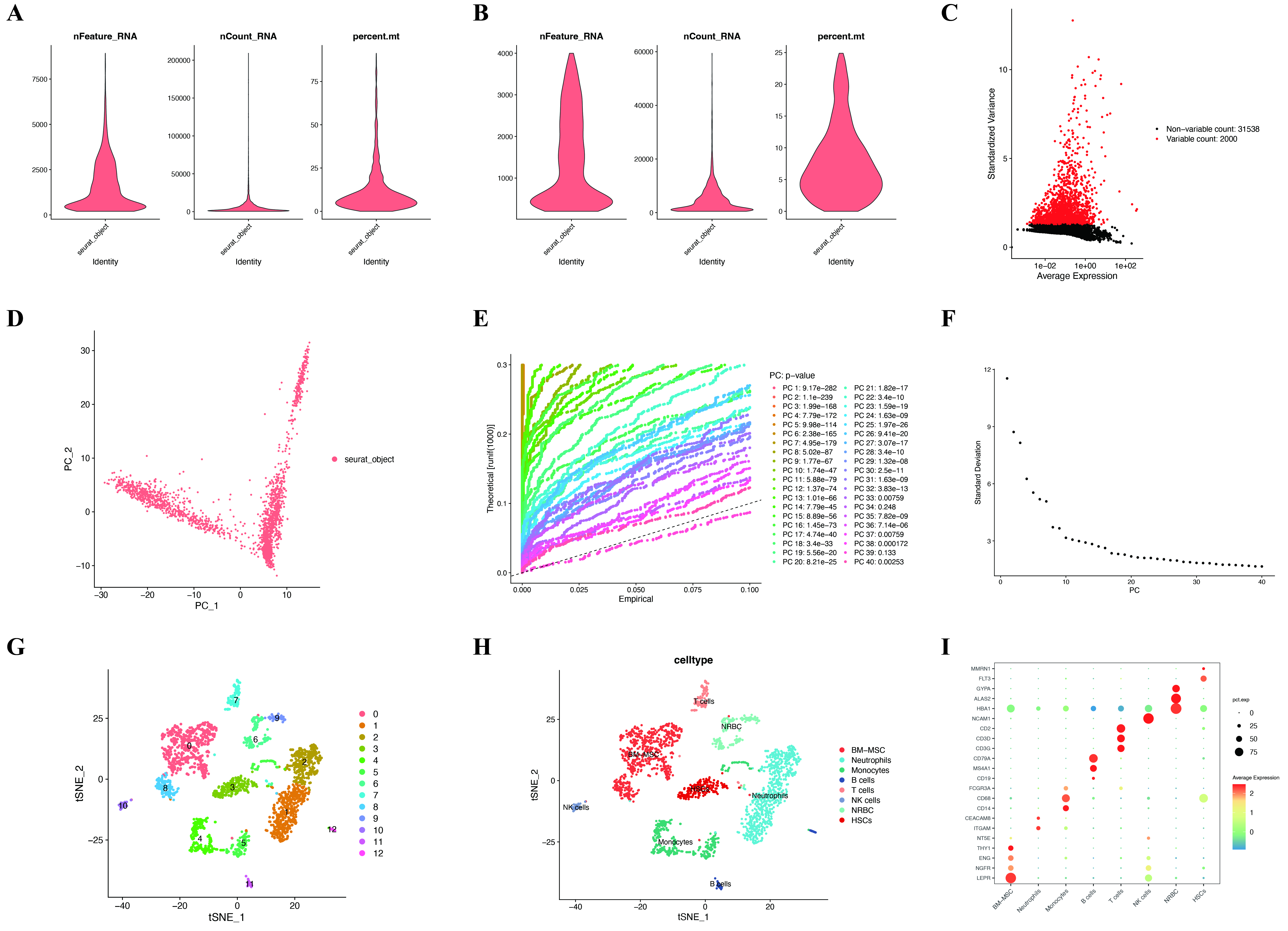

Supplement: Supplementary Figure 3 — Single-cell RNA sequencing quality control and dimensionality reduction analysis.​(A) Violin plots displaying raw cellular distributions of total genes, RNA counts, and mitochondrial gene percentage across all cells before quality control. (B) Post-filtering distributions showing retained high-quality cells. (C) Selection of top 2,000 highly variable genes for downstream analysis. (D) PCA plot of the first two principal components, illustrating variance distribution across cells after HVG-based dimensionality reduction. (E) Jackstraw analysis confirming significant PCs. (F) Elbow plot selecting 20 PCs for t-SNE clustering. (G) t-SNE visualization of 13 initial cell clusters. (H) ​Annotated cell types based on marker genes. (I) Dot plot of cell type-specific marker expression. [file Image3.jpeg]

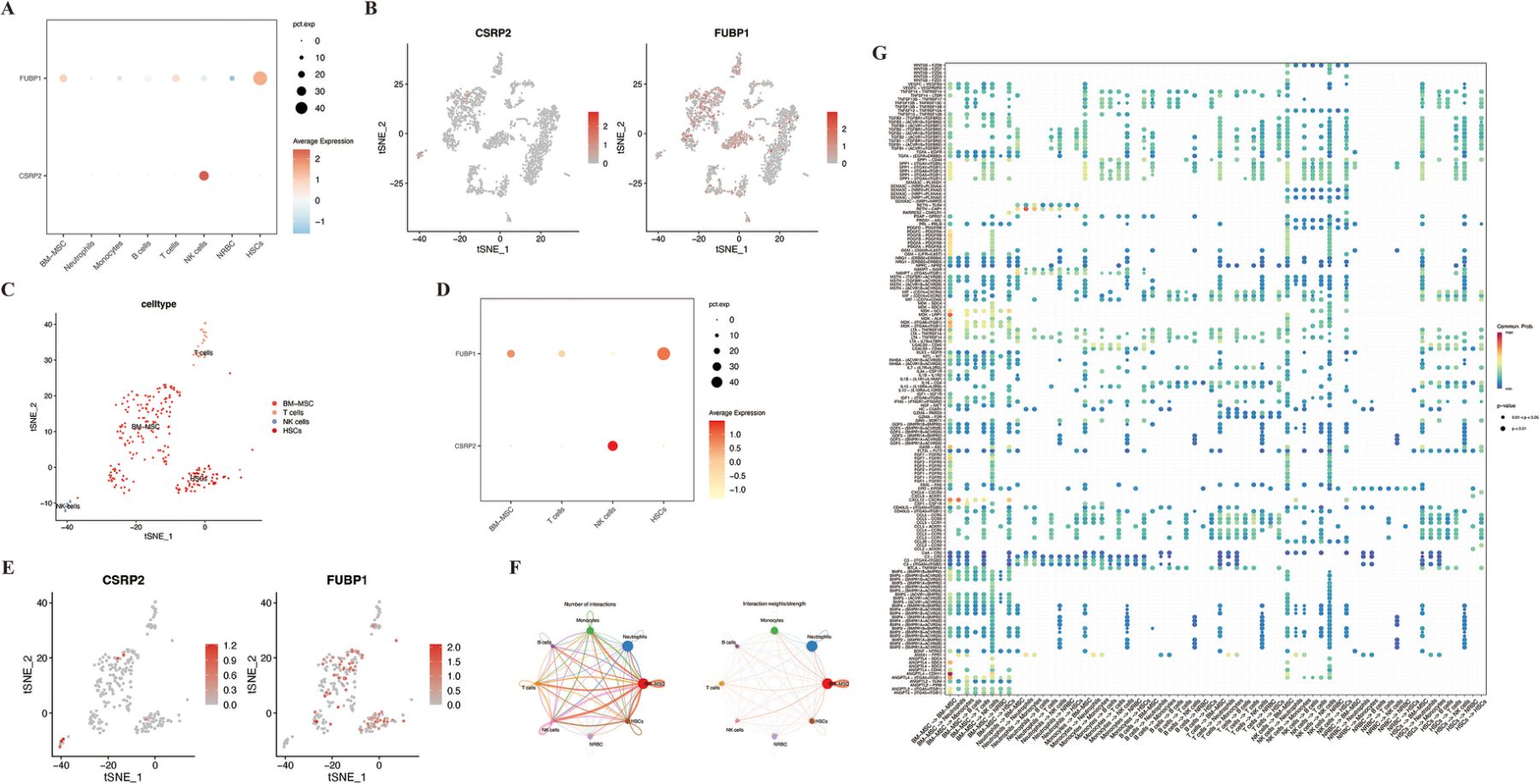

Supplement: Supplementary Figure 4 — Single-cell RNA sequencing reveals biomarker expression dynamics and intercellular communication in osteoporosis. (A, B) FeaturePlot visualization of CSRP2 and FUBP1 expression across 8 annotated cell types. CSRP2 exhibited predominant expression in NK cells, while FUBP1 was enriched in BM-MSCs, T cells, and HSCs. (C, D) t-SNE plots showing spatial distribution of key cell populations and biomarker expression patterns. (E) DotPlot quantification confirming elevated CSRP2 in NK cells and FUBP1 in HSCs. (F) Cell-cell communication network analyzed by CellChat, highlighting prominent interactions between BM-MSCs and NK cells. (G) Receptor-ligand pairs mediating intercellular crosstalk. [file Image4.jpeg]

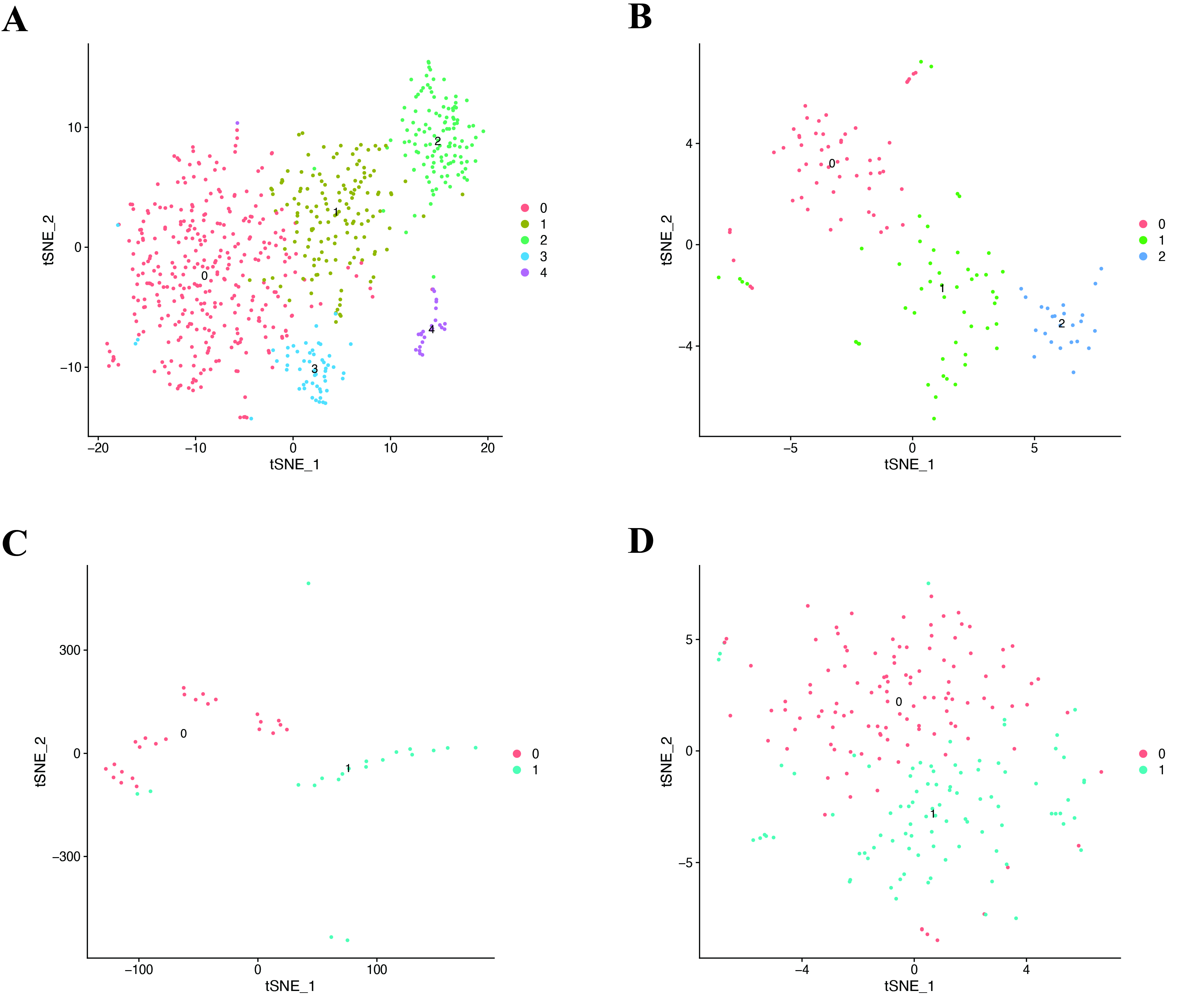

Supplement: Supplementary Figure 5 — Secondary dimensionality reduction and clustering of key cell populations in osteoporosis bone marrow. (A) BM-MSCs cells subdivided into 5 distinct subtypes via t-SNE clustering. (B) T cells segregated into 3 transcriptional subtypes. (C) NK cells partitioned into 2 subpopulations. (D) HSCs resolved into 2 heterogeneous clusters. [file Image5.jpeg]

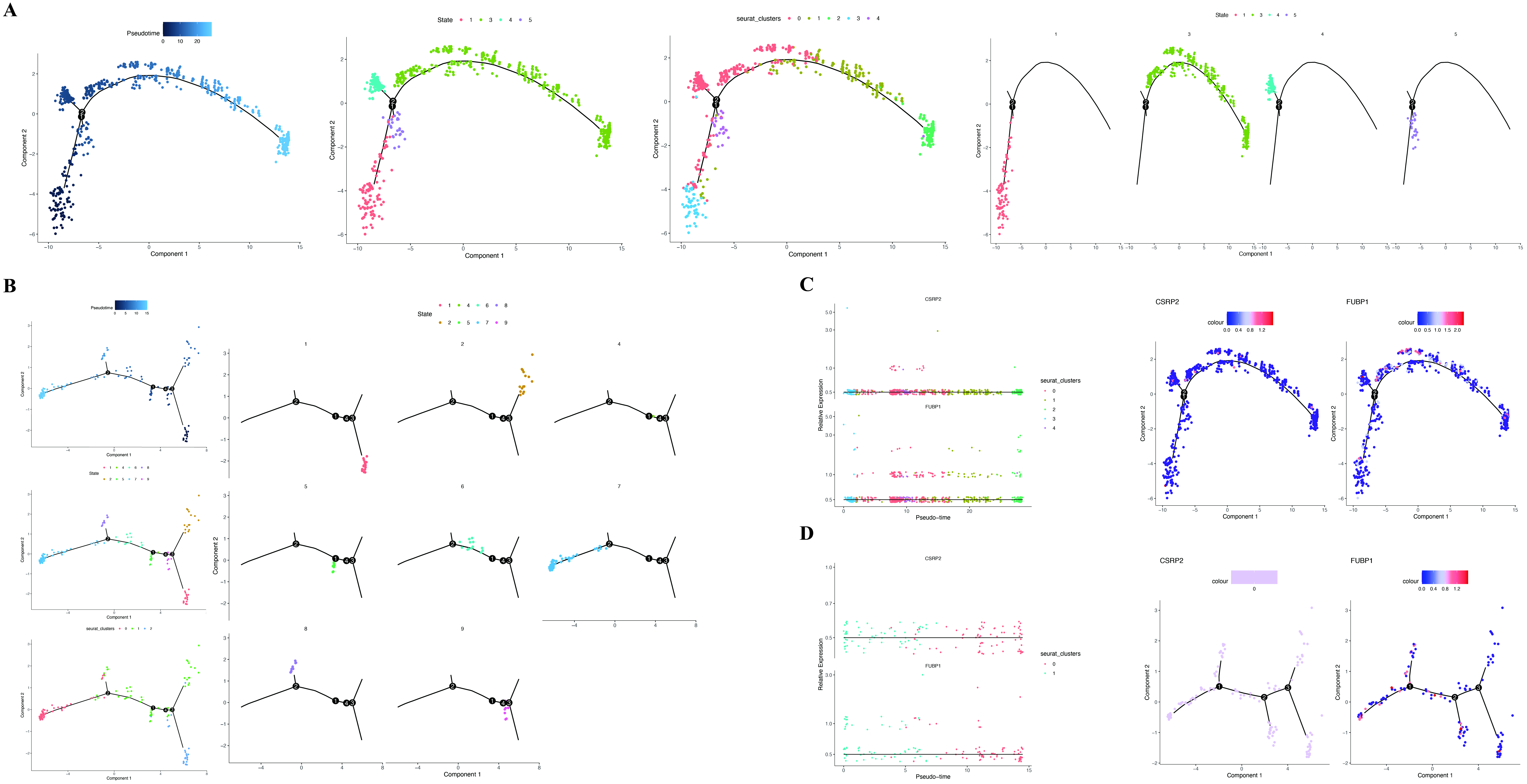

Supplement: Supplementary Figure 6 — Differentiation trajectories and biomarker expression dynamics in BM-MSCs and T cells. (A) Pseudotime trajectory analysis of BM-MSCs showing differentiation states. (B) T cell pseudotime trajectory with differentiation progression. (C, D) Heatmaps depicting conserved expression patterns of CSRP2 and FUBP1 across BM-MSC (C) and T cell (D) differentiation states. [file Image6.jpeg]

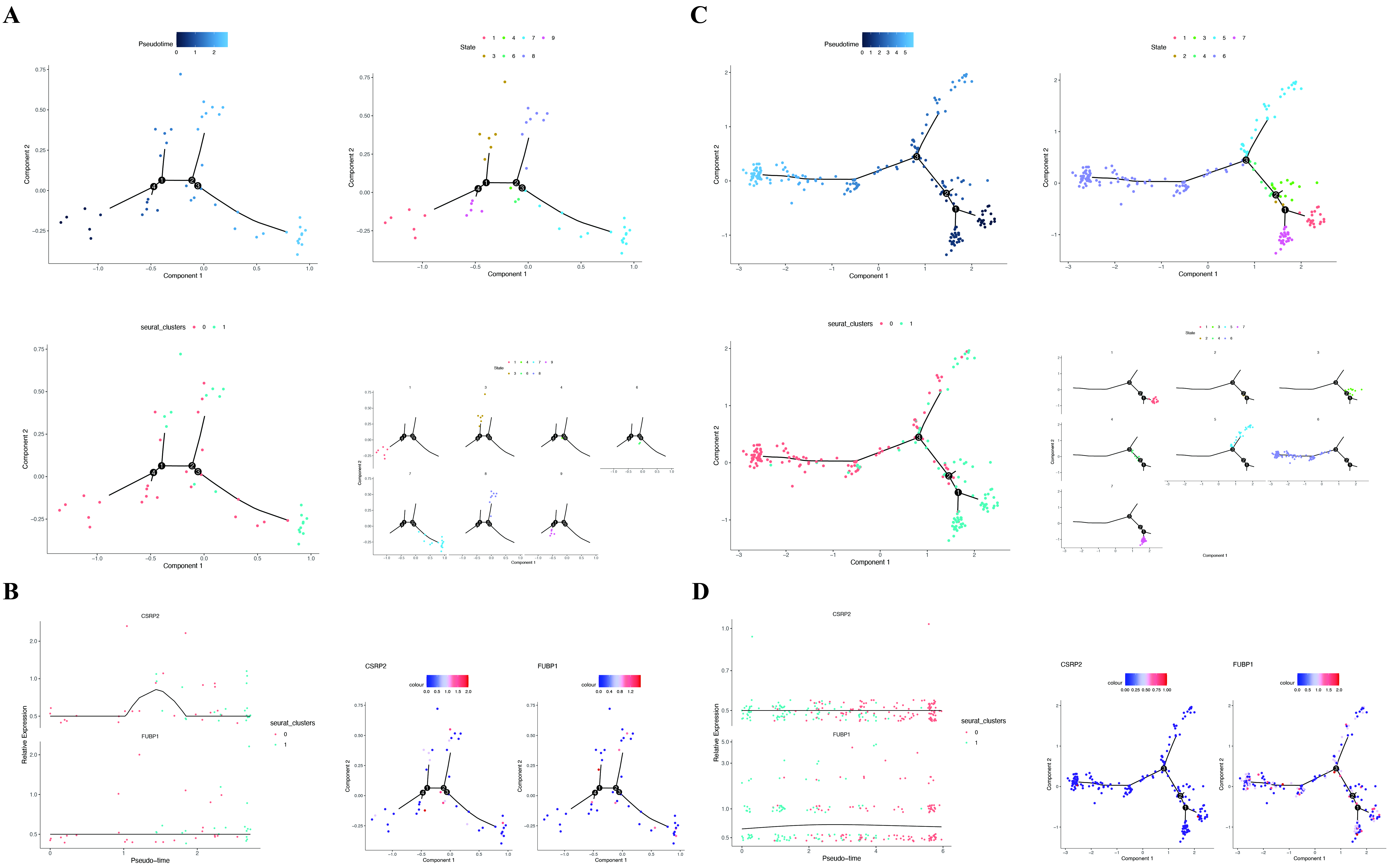

Supplement: Supplementary Figure 7 — Pseudotime trajectory analysis reveals dynamic biomarker expression during NK cell and HSC differentiation.​(A) t-SNE visualization of NK cell differentiation states, with pseudotime trajectory. Subtype 0 represents earlier differentiation stages. (B) Expression dynamics of CSRP2 and FUBP1 across NK cell pseudotime. (C) t-SNE plot of HSC differentiation states, with pseudotime trajectory. Subtype 1 marks earlier differentiation. (D) FUBP1 expression gradually increases during HSC differentiation before slight decline, while CSRP2 remains conserved. [file Image7.jpeg]
