# Supplementary material for: Identification of lactylation-related biomarkers in osteoporosis from transcriptome and single-cell data
Source: Front Endocrinol (Lausanne). 2025 Aug 25;16:1621878. doi: 10.3389/fendo.2025.1621878 (PMC12414738; doi:10.3389/fendo.2025.1621878)
Supplement: Supplementary Table 2 — ​Marker Genes for Annotated Cell Types in scRNA-seq Analysis. [file Table2.docx]

**Supplementary Table 2 Cell types and their marker genes**

| Cell types | Marker genes |
| --- | --- |
| Bone marrow mesenchymal stem cells (BM−MSCs) | LEPR, NGFR, ENG, THY1, NT5E |
| Neutrophils | ITGAM, CEACAM8 |
| Monocytes | CD14, CD68, FCGR3A |
| B cells | CD19, MS4A1, CD79A |
| T cells | CD3G, CD3D, CD2 |
| Natural killer (NK) cells | NCAM1 |
| nucleated red blood cells (NRBCs) | HBA1, ALAS2, GYPA |
| Hematopoietic stem cells (HSCs) | FLT3, MMRN1 |
