# Supplementary material for: Identification of lactylation-related biomarkers in osteoporosis from transcriptome and single-cell data
Source: Front Endocrinol (Lausanne). 2025 Aug 25;16:1621878. doi: 10.3389/fendo.2025.1621878 (PMC12414738; doi:10.3389/fendo.2025.1621878)
Supplement: Supplementary Table 3 — Primer Sequences for RT-qPCR Validation of Biomarkers and GAPDH​. [file Table3.docx]

**Supplementary Table 3 Primer sequences for biomarkers and GAPDH**

| **Primer** | **Sequences** | |
| --- | --- | --- |
| CSRP2 F | TGGGAGGACCGTGTACCAC | |
| CSRP2 R | CCGTAGCCTTTTGGCCCATA |  |
| FUBP1 F | CAACCAGATGCTAAGAAAGTTGC |  |
| FUBP1 R | CCTCCTCTGCCAATTATGAATCC |  |
| GAPDH F | ATGGGCAGCCGTTAGGAAAG |  |
| GAPDH R | AGGAAAAGCATCACCCGGAG |  |
